# Supplementary material for: Integration of Network Pharmacology, Molecular Docking, and In Vitro Nitric Oxide Inhibition Assay to Explore the Mechanism of Action of Thai Traditional Polyherbal Remedy, Mo-Ha-Rak, in the Treatment of Prolonged Fever
Source: Pharmaceuticals (Basel). 2025 Oct 13;18(10):1541. doi: 10.3390/ph18101541 (PMC12567552; doi:10.3390/ph18101541)
Supplement: Supplementary file 1 [file pharmaceuticals-18-01541-s001.zip › pharmaceuticals-3892874-supplementary.pdf]

Supplementary Materials

Integration of Network Pharmacology, Molecular Docking and In Vitro Nitric Oxide Inhibition Assay to Explore the Mechanism of Action of Thai Traditional Polyherbal Remedy, Mo-Ha-Rak, in the Treatment of Prolonged Fever

Chinnaphat Chaloemram [1], Ruchilak Rattarom [2], Anake Kijjoa [3] and Somsak Nualkaew [2,*]

[1] Doctor of Philosophy in Pharmacy program, Faculty of Pharmacy, Mahasarakham University, Kantharawichai, Maha Sarakham 44150, Thailand; chinnaphat.med@gmail.com (C.C.)

[2] Pharmaceutical Chemistry and Natural Product Research Unit, Faculty of Pharmacy, Mahasarakham University, Kantharawichai, Maha Sarakham 44150, Thailand; rujiluk.r@msu.ac.th (R.R.); somsak.n@msu.ac.th (S.N.)

[3] School of Medicine and Biomedical Sciences (ICBAS) and CIIMAR, Universidade do Porto, Rua de Jorge Viterbo Ferreira 228, 4050-313 Porto, Portugal; ankijjoa@icbas.up.pt (A.K.)

* Correspondence: somsak.n@msu.ac.th (S.N.); Tel.: +665-137-6763

Table S1. ADME analysis of 147 anti-inflammatory bioactive compounds in MHR.

Anti-inflammatory bioactive compounds in MHR	GI absorption	Drug likeness
(+)-Vouacapenic acid	High	Yes
3-Methoxy quercetin	High	Yes
4-*O*-Methylgallic acid	High	Yes
5, 7-Dihydroxy-6-oxoheptadecanoic acid	High	Yes
Apigenin	High	Yes
Apigenin 6-C-β-D-glucopyranoside	Low	No
Apigenin 8-C-β-D-glucopyranoside	Low	No
Ascorbic acid	High	Yes
Astragalin (Kaempferol 3-*O*-glucoside)	Low	No
Augustic acid	High	Yes
Azadirachtin	High	Yes
Berberine	High	Yes
Bergapten	High	Yes
Bergenin	High	Yes
Borapetol B	High	Yes
Butylidenephthalide	High	Yes
Caffeic acid	High	Yes
Casuarinin	Low	No
Catechin	Low	No
Chebulagic acid	High	Yes
Chebulanin	Low	No
Chebulic acid	Low	No
Chebulinic acid	Low	No
Chlorogenic acid	Low	No
Chrysophanol	High	Yes

Citreorosein	High	Yes
Cnidilide	High	Yes
Columbin	High	Yes
Corilagin	Low	No
Crispinoid D	Low	No
Cycloartenol	Low	No
Daucosterol	Low	No
Diosmetin	High	Yes
Ellagic acid	High	Yes
Emodin	High	Yes
Epicatechin	Low	No
Epigallocatechin	Low	No
Ethyl gallate	High	Yes
Eugenol	High	Yes
Ferulic acid	High	Yes
Friedelin	Low	No
Furosin	High	Yes
Gallic acid	High	Yes
Gallocatechin	Low	No
Genkwanin	High	Yes
Genkwanin 7-glucoside	Low	No
Geraniin	High	Yes
Harperamone	High	Yes
Harperfolide	Low	No
Harrisolanol A	High	Yes
Harrisonin	Low	No
Higenamine	High	Yes
Hispidulin	High	Yes
Isoorientin (Luteolin-6-C-glucoside)	High	Yes
Isoquercetin	Low	No
Isorhamnetin	Low	No
Isorhamnetin-3-*O*-glucoside	Low	No
Isoscopoletin	High	Yes
Jatrorrhizine	High	Yes
Kaempferol	High	Yes
Kaempferol-3-*O*-glucuronide	Low	No
Kaempferol-3-*O*-robinobioside	Low	No
Ligustilide	High	Yes
Limonene	Low	No
Linalool	High	Yes
Loureirin A	High	Yes

Loureirin B	High	Yes
Loureirin C	High	Yes
Lupeol	Low	No
Lupeol acetate	Low	No
Luteolin	High	Yes
Luteolin 4′-methyl ether 7-glucoside	Low	No
Luteolin 6-C-β-D-glucopyranoside	Low	No
Luteolin 8-C-β-D-glucopyranoside	Low	No
Magnoflorine	High	Yes
Marrubiagenin-methylester	High	Yes
Methyl gallate	High	Yes
Myrcene	Low	No
Myricetin 3-*O*-galactoside	Low	No
Myricetin-3-*O*-glucoside	Low	No
Nimbin	High	Yes
Nimbolide	High	Yes
N-trans-feruloyltyramine	High	Yes
Obacunone	High	Yes
O-Methylalloptaeroxylin	High	Yes
Orientin (Luteolin-8-glucoside)	Low	No
Palmatine	High	Yes
p-Coumaric acid	High	Yes
Pectolinarigenin	High	Yes
Pedunculagin	Low	No
Perforatic acid	High	Yes
Perforatic acid methyl ester	High	Yes
Peucenin-7-methyl ester	High	Yes
Phloretin	High	Yes
Physcion	High	Yes
Protocatechuic acid	High	Yes
Pterostilbene	High	Yes
Punicalagin A	Low	No
Punicalagin B	Low	No
Pyrogallol	Low	No
Quercetin	High	Yes
Quercetin 3-*O*-galactoside	Low	No
quercitrin	Low	No
Racemosic acid	Low	No
Resveratrol	High	Yes
Rhein	High	Yes
Rhein methyl ester	High	Yes

Rutin	Low	No
Salsolinol	High	Yes
Secoisolariciresinol	High	Yes
Spathulenol	High	Yes
Stachydrine	Low	No
Stigmasterol	Low	No
Stigmasterol glucoside	High	Yes
Syringaresinol	High	Yes
Syringin	Low	No
Taraxerol	Low	No
Tinocrisposide	High	Yes
Tinopanoid A	Low	No
Tinopanoid B	Low	No
Tinopanoid C	High	Yes
Tinopanoid D	High	Yes
Tinopanoid E	High	Yes
Tinopanoid F	High	Yes
Tinopanoid G	High	Yes
Tinopanoid H	Low	No
Tinopanoid I	Low	No
Tinopanoid J	Low	No
Tinopanoid M	High	Yes
Tinopanoid R	High	Yes
Tinospin C	High	Yes
Tinosporol C	High	Yes
Tinotufolin D	High	Yes
Tyramine	High	Yes
Valencene	Low	No
Vanillic acid	High	Yes
Vitexin	Low	No
Ziganein	High	Yes
α-Amyrin	Low	No
α-Amyrin acetate	Low	No
α-Pinene	Low	No
α-Santalol	High	Yes
β-Amyrin	Low	No
β-Caryophyllene	Low	No
β-Pinene	Low	No
β-Santalol	High	Yes
β-Sitosterol	Low	No

Table S2. The 965 targets associated with 86 bioactive compounds.

Anti-inflammatory compounds in MHR	Predicted targets
(+)-Vouacapenic acid	TBXAS1, POLA1, PIK3CA, PIK3R1, HSD11B1, TBXA2R, PTGES2, PTGDR2, PTGDR, PTGES, MME, MDM2, PSEN2, PSENEN, NCSTN, APH1A, PSEN1, APH1B, PTGER4, PTGER2, PPARG, PTGER3, PTGS2, ALOX5, PTGS1, F2R, SLC22A12, ACE, PTGER1, PTGIR, FDFT1
3-Methoxy quercetin	XDH, CA2, CA7, CA12, CA4, CYP1B1, ABCC1, NOX4, AKR1B1, ABCG2, ACHE, ALOX15, ALOX12, IGF1R, EGFR, AVPR2, MAOA, FLT3, CYP19A1, F2, PIM1, ALOX5, AURKB, DRD4, ADORA1, GLO1, MPO, PIK3R1, ADORA2A, DAPK1, PYGL, CA1, GSK3B, SRC, PTK2, HSD17B2, KDR, MMP13, MMP3, CA3, PLK1, CA6, CDK1, MMP9, MMP2, PKN1, CA14, CA9, CSNK2A1, MET, NEK2, CXCR1, CAMK2B, ALK, AKT1, ABCB1, NEK6, PLA2G1B, CA5A, BACE1, AXL, NUAK1, AKR1C2, AKR1C1, AKR1C3, AKR1C4, CA13, AKR1A1, GPR35, MAPT, KDM4E, TOP2A, INSR, MYLK, SYK, PIK3CG, APEX1, CDK5R1, CDK5, CCNB3, CDK1, CCNB1, CCNB2, CDK6, CDK2, ARG1, APP, MCL1, TERT, TYR, HSD17B1, AHR, ESRRA, PTPRS, PLG, ESR2, MPG, SLC22A12, PARP1, TTR, MMP12, CD38, AKR1B10, TNKS2
4-*O*-Methylgallic acid	CA2,CA7, CA1, CA3, CA6, CA12, CA14, CA9, CA5A, FUT7, TPMT, CA4, TTR, CA13, CA5B, SQLE, SERPINE1, TUBB1, POLA1, POLB, IGF1R, ALK
5, 7-Dihydroxy-6-oxoheptadecanoic acid	PTGER2, PTGFR, FFAR1, SLC22A6, PPARA, PPARD, CHRNA7, HMGCR, G6PD, FDFT1, FNTA, FNTB, FABP4, FABP3, FABP5, NR1H4, AKR1B10, HSD11B1, PTGER1, PTGER4, PTGIR, UGT2B7, GPBAR1, HAO1, SERPINA6, SHBG, HSD17B3, GABBR1, NPC1L1, PTGER3, PTGDR, VDR, FABP2, PDE6D, MME, CDC25A, GABRA2, GABRB2, GABRG2, TBXAS1, KDM2A, EDNRA, MAPK14, MAPK10, MMEL1, ITGB1, ITGA4, GCGR, GIPR, CYP2C9, CA2, CTSA, CASP3, CASP6, CASP7, CASP8, CASP1, PTGDR2, EDNRB, PDE5A, EGLN1, P2RY12, P2RX3, PHF8, GSK3B, GSK3A, PGR, BACE1, SLC5A2, PLA2G4B, TBXA2R, SCN9A, KDM5C, MMP1, THRA, THRB, DUSP23, DUSP15, PTPN22, PTPN13, PTPN12, PTPN11, PTPN9, GLP1R, TYMS, PDE4B, DGAT1, IDE, AGTR1, APP, CSNK2A1, ITGAV, ITGB3, EP300, SCN10A, FOLH1, SOAT1, F5, CYP19A1, TNF, SLC6A1, MMP9, MMP2, MMP8
Apigenin	NOX4, AKR1B1, CDK5R1, CDK5, XDH, MAOA, FLT3, CYP19A1, ESR1, CCNB3, CDK1, CCNB1, CCNB2, ACHE, ADORA1, PTGS2, ESR2, CDK6, ADORA2A, SYK, GSK3B, ABCC1, HSD17B1, TTR, CSNK2A1, CFTR, CYP1B1, ABCG2, AKR1B10, TNKS2, TNKS, ALOX5, PARP1, CA2, CA7, CA12, ABCB1, CA4, ALOX12, PTPRS, GLO1, APP, MMP9, MMP2, MMP12, CD38, TOP1, ARG1, ESRRA, PFKFB3, ALOX15, AMY1A, GRK6, TYR, HSD17B2, AHR, CA1, CA9, CBR1, AR, TERT, PIM1, EGFR, CDK1, KDM4E, LCK, AURKB, NAE1, TBXAS1, IGF1R, KDR, PLK1, MET, ALK, AXL, BCHE, ADORA3, CDK2, HTR2C, GPR35, DAPK1, MPG, SLC22A12, ST6GAL1, F2, PLG, AVPR2, DRD4, MPO, PIK3R1, PYGL, SRC, PTK2, MMP13, MMP3, CA3, CA6, PKN1, CA14, NEK2, CXCR1, CAMK2B, AKT1, NEK6
Ascorbic acid	GSK3B

Augustic acid	PTPN1, AKR1B10, HSD11B1, POLB, PDE4D, PTPN2, PLA2G1B, CDC25B, RORC, PTPRF, ACP1, CD81, NOS2, PTGES, CES2, FNTA, FNTB, AR, PTPN6, FABP1, SCD, PPARG, PTGS2, HSD11B2, PRKCH, PTPN11, PPARA, PPARD, ESR2, CYP19A1, PREP, TERT, NR3C2, FAAH, FABP4, FABP3, FABP5, CDC25A, SERPINA6, SHBG, G6PD, CYP51A1, PTGS1, LTB4R, SIGMAR1, CYP17A1, HMGCR, PTGER2, ADORA3, NR1H3, TLR9, ALOX5AP, NR3C1, TOP1, PGR, TOP2A, ALOX5, SRD5A2, PTGDR2, BCHE, NR1H4, PTGER4, SLC10A2, SLC10A1, FFAR1, ESR1, GRIK1, CHRM2, SLC6A2, GRIK2, GPBAR1, PTGIR, BACE1, RORA
Azadirachtin	GLI1, ADORA1, P2RX3, MCHR1, FDFT1
Berberine	ACHE, HTR2B, BCHE, ADRA2C, ADRA2B, CHRM1, SIGMAR1, CYP2D6, SAE1, UBA2, RAC1, CDC42, RPS6KB1, AURKA, AURKB, CYP11B2, PRF1, GRIA1, TBXAS1, HPGD, SLC1A3, GABRB3, GABRA3, GABRG2, GABRB3, GABRG2, GABRA1, GABRB3, GABRG2, GABRA5, PIM1, PIM2, MET, HTR3A, GRK5, CYP11B1, IMPDH2, PIK3CA, PIK3R1, F3, CYP19A1, BCAT2, TRPM8, ICAM1, SELE, MAOB, PIK3CD, PIK3CB, PIK3CG, MAPKAPK2, PARP10, CHEK2, CCNC, CDK8, CDK8, MAPK10, AGPAT2, TGM2, SCD, XBP1, TYMS, DHFR, GABRA2, GABRB3, GABRG2, ROCK1, PRKACA, CHRM4, JAK2, LCK, CDK9, PTPN1, ALOX5AP, CHEK1, KIT, SRC, MKNK1, IKBKB, MAPK14, LRRK2, ROCK2, ATR, AOC3, GRK3, PLK1, GRK2, NPY5R, MAP4K4, NTRK1, ABL1, CDK1, CCNB1, NR3C2, PGR, DRD4, PTGS2, DRD3, CDK2, CDK4, SIRT2, ADORA2A, ADORA3, GRM5, QPCT, CSF1R, CNR2, CBFB, HSD17B1, PNMT, PARP2, PTGES, RPS27
Bergapten	CBR1, KCNA5, KCNA3, CA12, CA9, CA7, CA13, CA1, ALOX5, ACHE, CYP1A2, CA6, CA14, CA4, CA5A, XDH, SRD5A1, CA5B, AKR1C3, AKR1C1, BACE1, MAOA, ESR2, PARP1, PARP2, NFKB1, GPR35, AKR1B1, CCND1, CDK4, PDGFRB, FLT4, INSR, PTK2, PLK1, MET, PLK4, TEK, MAP3K8, BRAF, EPHB4, HSPA1A, NUAK1, SQLE, FGR, LYN
Bergenin	F10, PNP, ADA, GSK3B, ADORA2A, ADORA3, ADK, CA14, TDP1, TYMP, GAPDH, MMP3, MMP9, MMP1, ADAM17
Borapetol B	OPRK1, OPRM1, OPRD1, NR3C1, MMP9, ALOX5, BRD4, CREBBP, AKR1C3, EIF2AK3, PDE10A, CDK2, BACE2, BACE1, SERPINA6, ADAM17, TTL, SLC5A4, SLC5A1, CDK1, ALPL, DPP4, EGFR, P2RX7, SORD, IL6, APP, GLUL, GPBAR1, FABP1, IMPDH1, IMPDH2
Butylidenephthalide	TRPM8, KCNK2, SLC6A3, CA2, CA1, SLC6A4, CHRM4, CHRM5, CHRM2, CHRM1, CHRM3, F2, PRSS1, CCR1, CCR5, CCR8, ALDH2, ELANE, CTSK, CYP11B1, GABRA2, GABRB2, GABRG2, CYP11B2, PDE10A, TGFBR1, P2RX7, ADRA2A, ADRA2C, ADRA2B, PTGS1, CTSH, PTGS2, CTSL, CTSB, CYP17A1, EPHX1, ADRA1A, TRPA1, PIK3CD, PIK3R1, GABRB3, GABRG2, GABRA5, ALOX15, MAOA, KCNH2, HSD11B1, GRM5, MET
Caffeic acid	CA2, ALOX5, CA7, CA1, CA6, MMP9, CA12, MMP1, MMP2, PTPN1, CA14, CA9, CA5B, CA5A, CA3, AKR1B1, ESR2, CA4, AKR1B10, HCAR2, MIF, CA13, NQO2, TLR4, ERBB2, ESR1, SLC6A2, TTR, MAPK1, AKR1C3, AKR1C4, AKR1C2, SYK, APP, EGFR, FYN, LCK, PTGS1, PIK3CB, CYP1A2, CYP2C9, CYP3A4, CYP2C19, PIK3CA, ELANE, F3, HSD11B1, NFE2L2, STAT3
Chebulagic acid	TOP1, SQLE, F10

Chrysophanol	ELANE, ESR2, CSNK2A1, PTP4A3, PIM1, ESR1, FTO, MCL1, BCL2, CYP19A1, FNTA, FNTB, EGFR, LIMK1, LCK, LDHB, ERN1, CISD1, DUSP3, CA7, CA14, PLEC, CSNK1A1, CSNK1D, LDHA, MMP16, MMP13, MMP3, MMP9, MMP1, MMP2, MMP14, MMP12, MMP8, HTR2B, HMGCR, PIK3CG, PLAA, HDAC6, HDAC8, HDAC1, HDAC5, HDAC7, CTSV, HDAC4, GUSB, ADAM17, MMP7, MAP2K1, NOX4, PLA2G7, ABL1, SRC, KDR, MTOR, PIK3CD, PRKDC, PIK3CB, HCK, PI4KB, PIK3CA, CHEK1, NOS2, MAOB, GRM5, ALPG, ALPL, HSP90AA1, HSP90AB1, DAO, EIF2AK2, ACE
Citreorosein	ESR1, ESR2, PIM1, CSNK2A1, PTP4A3, FTO, ELANE, CYP19A1, FNTA, FNTB, MCL1, LIMK1, BCL2, LDHA, LDHB, NOX4, CDK5R1, CDK5, XDH, FLT3, CCNB3, CDK1, CCNB1, CCNB2, GLO1, APP, SYK, GSK3B, PARP1, TTR, MMP9, MMP2, MMP12, CD38, CYP1B1, ABCG2, AKR1B10, TNKS2, TNKS, TOP1, ADORA1, ARG1, PTPRS, PLK1, CDK6, CDK2, DAPK1, SLC22A12, PPARG, ABCC1, AHR, ESRRA, ABCB1, F2, DRD4, MPO, PIK3R1, PYGL, MMP13, MMP3, CDK1, PKN1, CAMK2B, AKT1, NEK6, PLA2G1B, BACE1, NUAK1, AKR1C2, AKR1C3, AKR1C4, AKR1A1, LCK, CASP3, CFTR, TBXAS1, MGAM, HTR2C, ESRRB
Cnidilide	CYP19A1, F2, PRSS1, CYP17A1, SLC6A3, FNTA, FNTB, CTRC, HMGCR, PTPN1, ACHE, GSK3B, EPAS1, HSD11B1, CA2, CA1, TNF, CES2, SLC6A2, PLA2G2A, PTPN2, EPHX1, ADRA2A, ADRA2C, ADRA2B, ADRA1A, KCNK2, AR, NOS2, TACR2, CTSK, CTSL, CTSB, LIPE, PTGS2, CTRB1, TAAR1, PDE10A, CTSH, CYP11B1, GABRA2, GABRB2, GABRG2, CYP11B2, PGR, ELANE, CREBBP, TBXAS1, EGFR, GABRB3, GABRG2, GABRA5, ICAM1, VCAM1, SELE, PGGT1B, FNTA, CHRNA4, USP10, USP13, P2RX7, DNTT, HRH3, HRH4, PRKDC, ADRA1D, HTR2C, PIK3CD, PIK3R1, PLA2G6, TNKS2, FLT3, ABCG2, CHRM1, HSD17B3, HTT, POLA1, POLB, GABRB3, GABRA3, GABRG2, GABRB3, GABRG2, GABRA1, GABRA2, GABRB3, GABRG2, GRM5, ALDH2, ALDH1A1, ALDH3A1, QPCT, NLRP3, TBXA2R, CCNB3, CDK1, CCNB1, CCNB2, FAAH, CYP2A6, NR3C2, ADORA2A, GRM4, XPO1, CHRM2
Columbin	OPRK1, OPRM1, ADORA2A, JAK1, LRRK2, GABRB3, GABRA3, GABRG2, GABRB3, GABRG2, GABRA1, GABRB3, GABRG2, GABRA5, GABRA2, GABRB3, GABRG2, MAP2K1, MET, MMP9, MMP1, MMP2, MMP8, OPRD1, HDAC6, HDAC1, TTL, POLA1, NR3C1, JAK2, ALOX5, ROCK2, ROCK1, PGR, CYP19A1, CCNC, CDK8, CDK8, KIT, CCR1, KDR, PDE10A, PIM1, JAK3, TYK2, SERPINA6, MDM2, PSEN2, PSENEN, NCSTN, APH1A, PSEN1, APH1B, PTPRF, ERN1, RET, LCK, MAPK1, MKNK2, ALK, EPHB4, IMPDH1, IMPDH2
Diosmetin	ABCC1, CYP1B1, XDH, CA2, CA7, CA12, CA4, AKR1B1, CDK5R1, CDK5, CCNB3, CDK1, CCNB1, CCNB2, ARG1, PLG, PTPRS, ABCB1, APP, NOX4, MAOA, FLT3, ALOX5, ADORA1, GLO1, GSK3B, MMP9, MMP2, ABCG2, SYK, PARP1, TTR, MMP12, CD38, AKR1B10, TNKS2, TNKS, TOP1, PIM1, ADORA2A, ACHE, CDK6, CYP19A1, PLA2G2A, TERT, ESR1, ESR2, CSNK2A1, HSD17B1, CBR1, OPRD1, IGF1R, EGFR, ALOX15, ALOX12, HSD17B2, CA1, CA9, KIT, CDK2, F2, CDK1, PTGS2, CFTR, AVPR2, AURKB, DRD4, MPO, PIK3R1, DAPK1, PYGL, SRC, PTK2, KDR, MMP13, MMP3, CA3, PLK1, CA6, PKN1, CA14, MET, NEK2, CXCR1, CAMK2B, ALK, AKT1, NEK6, PLA2G1B, CA5A, BACE1, AXL, NUAK1, AKR1C2,

	AKR1C1, AKR1C3, AKR1C4, CA13, AKR1A1, MCL1, GPR35, ST6GAL1, AMY1A, GRK6
Ellagic acid	F10, HSP90AB1, IDO1, FOLH1
Emodin	ESR1, PIM1, ESR2, CSNK2A1, PTP4A3, ELANE, FNTA, FNTB, MCL1, BCL2, FTO, LIMK1, LCK, CYP19A1, ABCB1, BCHE, XDH, ADORA3, CYP1B1, AURKB, KDR, PLK1, MET, AXL, EGFR, FASN, PARP1, TNKS2, TNKS, CRHR1, DRD3, AKR1B1, CDK5R1, CDK5, CCNB3, CDK1, CCNB1, CCNB2, CDK6, ABCG2, CBR1, TBXAS1, MGAM, HTR2C, ESRRA, ESRRB, LDHA, LDHB, NOX4, FLT3, SYK, GSK3B, ABCC1, TTR, CFTR, AKR1B10
Ethyl gallate	SQLE, CA2, CA7, CA1, CA12, CA14, CA9, FUT7, CA13, CA4, SERPINE1, CA3, CA5A, ESR2, IGF1R, ALK, AURKB, SRC, PTK2, KDR, MET, NEK2, AXL, CA6, CA5B, BCL2L1, TYR, ADAMTS5, SNCA
Eugenol	FADS1, HDAC6, EGLN1, VEGFA, CA2, GPR84, PTGS1, DAO, PARP1, SRC, ADORA1, ADORA2A, SRD5A1, CHRNA3, CHRNA4, CXCR2, DCTPP1, ALPL, METAP2, CBR1, KCNMA1, ALOX15, CHRNA3, CHRNB4, KDR, HDAC8, AR, PSMB5, TDO2, CHRNA4, CHRNB2, HSD17B3, CYP17A1, AURKA
Ferulic acid	CA2, CA7, CA1, CA6, CA12, CA14, CA9, CA5A, CA5B, ALOX5, MMP9, MMP1, MMP2, PTPN1, MAOB, CA13, AKR1B1, CA3, APP, NFE2L2, STAT3, HSD11B1, ESR2, CA4, TLR4, PTGS1, MET, CYP1A1, CYP1A2, NQO2, CYP1B1, EGFR, PTGS2, TTR, TUBB1, RELA, FYN, LCK, ADORA1, ADORA2A, ADORA2B, TLR9, AKR1B10, ALOX15, PRKCE, F3, NOS2, SLC16A1, CCND1, CDK4, TUBB3, ABCB1, CPA1, FBP1, TOP2A, GLO1, BACE1, PARP1, KDM4C, AHR, AMPD3
Furosin	SQLE, ABCB1
Gallic acid	CA2, CA7, CA1, CA3, CA6, CA12, CA14, CA9, FUT7, CA4, CA5B, CA5A, CA13, SQLE, LDHA, LDHB, TTR, IGF1R, ALK, SERPINE1, ESR2, BCL2L1, GPR35, COMT, TPMT
Genkwanin	ADORA1, ADORA2A, ESR2, PIM1, ESR1, ABCB1, AKR1B1, HSD17B1, CYP1B1, FLT3, ABCG2, PTGS2, CDK5R1, CDK5, XDH, CYP19A1, CCNB3, CDK1, CCNB1, CCNB2, CDK6, NOX4, ABCC1, PLG, PTPRS, AMY1A, GRK6, TNKS2, TNKS, MAOA, ACHE, SYK, GSK3B, TTR, CSNK2A1, CFTR, AKR1B10, CA2, CA1, CA12, CA9, AR, CA7, CA4, CBR1, TERT, SLC22A12, APP, HSD17B2, ADORA3, KIT, OPRD1, EGFR, KDM4E, ALOX15, CDK1, ALOX12, NOS2, ALOX5, MCL1, NAE1, PARP1, LCK, PLA2G2A, SIGMAR1, PFKFB3, IGF1R, ARG1, GLO1, MMP9, MMP2, MMP12, CD38, TOP1, PIK3CG, TYR, AHR, ESRRA, ODC1, ST6GAL1, F2, CALM1, KDM5A, PPARG, CYP1A1, CYP1A2, PTPN1, MET, OPRM1, IKBKB, NTRK2, BACE1, AURKB, SRC, MAOB, PDE4D, GPR35, DAPK1, MPG, BCHE, FYN, TACR2, PRKDC, MAPK3
Geraniin	SQLE, TOP1
Harperamone	BCL2, RET, MAPK1, EPHB4, MCL1, HSP90AA1, ABCC9, MMP3, MMP13, MMP1, ADAM17, CA5A, PSEN2, PSENEN, NCSTN, APH1A, PSEN1, APH1B, PRKCB, GABRB3, GABRA3, GABRG2, GABRB3, GABRG2, GABRA1, GABRB3, GABRG2, GABRA5, GABRA2, GABRB3, GABRG2,

	GABRG2, GABRB3, GABRA6, MAP2K1, MPO, PDE4B, TTK, MAPK14, JAK1, CHEK1, TKT, PDE2A, PRKDC, PDE10A, BRD4, BRD2, BRD3, MME, MMP14, EIF2AK3, ABCB1, COMT, MTOR, PIK3CD, PIK3CB, MMP8, TNK2, PIK3C2B, NTRK1, KIF11, PDE3A, NQO2, MMP7, ERN1, CREBBP, PDE5A, ADA, NR3C1, CXCR2, PTAFR, CXCR1, HSD11B1, PDE7A, CYP17A1, TLR8, TLR7, ALOX5, OPRK1, HMGCR, EDNRA, CACNA1C, SRC, GRM5, HCK, SCN9A, PAK1, CASP8, CASP1, CYP2D6, PRKCG, CX3CR1, CDC7, KDM1A, ASAH1, IMPDH2, EWS-Fli1, CCR5, KCNH2, PI4KB, MDM2, MAPKAPK2, TYMP, BAZ2B, FCER2, BAZ2A, CYSLTR1, ELAVL1, EDNRB, ADRA2A, GYS1, F2, PLEC, MAP3K12, PSEN1
Harrisolanol A	-
Higenamine	DRD2, DRD4, ADRB2, ADRB1, DRD3, DRD1, TBXA2R, SIGMAR1, SLC6A3, DRD5, ADRA1D, ADRA1A, ADRB3, PRCP, ADRA1B, SLC6A4, OPRM1, OPRK1, ESR2, PNMT, DHCR7, CHRM4, BCHE, ACHE, KCNN1, KCNN3, KCNN2, OPRD1, F3, SLC6A2, HTR1A, ESR1, RBBP9, ADRA2A, ADRA2C, ABCB1, HCRTR2, HCRTR1, MAOA, MTNR1B, OPRL1, SLC47A1, CHRNA3, CHRNB4
Hispidulin	PIM1, ADORA1, ADORA2A, AKR1B1, PTPRS, KIT, OPRD1, FLT3, ESR2, AMY1A, GRK6, NOS2, NOX4, ESR1, HSD17B1, EGFR, CYP1B1, CDK5R1, CDK5, APP, TERT, ABCC1, CBR1, AR, XDH, PFKFB3, ALOX5, KDM4E, ALOX15, CDK1, ALOX12, ABCG2, HSD17B2, ADORA3, ABCB1, TNKS2, TNKS, MAOA, CYP19A1, CCNB3, CDK1, CCNB1, CCNB2, ACHE, PTGS2, CDK6, SYK, GSK3B, TTR, CSNK2A1, CFTR, AKR1B10, CA2, CA7, CA12, CA4, PLG, PLA2G2A, SLC22A12, CA1, CA9, F2, NAE1, ODC1, MCL1, GLO1, PARP1, MMP9, MMP2, MMP12, CD38, TOP1, ARG1, BACE1, TYR, AHR, ESRRA, CALM1, MAOB, IGF1R, ST6GAL1, OPRM1, LCK, SIGMAR1, DRD2, CYP1A1, CYP1A2, PTPN1, GPR35, DAPK1, MPG, PIK3CG, AURKB, SRC, PTK2, KDR, PLK1, PKN1, MET, NEK2, ALK, AKT1, NEK6, AXL
Isoorientin (Luteolin-6-C-glucoside)	AKR1B1, CA7, CA12
Isoscopoletin	CA12, CA9, CA13, CA7, CA1, CA14, CA5A, CA4, CA6, CBR1, CA5B, MAOA, ALOX5, EGFR, CA2, AKR1C1, SRD5A1, XDH, CDK2, CCNA1, CCNA2, KCNA3, MAOB, FLT4, INSR, PTK2, PLK1, PLK4, TEK, MAP3K8, HSPA1A, NUAK1, FGR, KCNA5, DAO, GSK3B, ESR2, KDR, AKR1C3, GSR, PTGS2, MB, SRC, PTPN1, APEX1, CDK9, CCNT1, PARP1, HSD17B3, ACHE, KCNMA1, CYP1A2, ALPG, PLAA, MET, CA3, ESR1, AOC3, CHRM1, NAT1, GPR35
Jatrorrhizine	ACHE, HTR2B, BCHE, ADRA2C, ADRA2B, CHRM1, SIGMAR1, CYP2D6, SAE1, UBA2, RAC1, CDC42, RPS6KB1, AURKA, AURKB, CYP11B2, PRF1, GRIA1, TBXAS1, HPGD, SLC1A3, GABRB3, GABRA3, GABRG2, GABRA1, GABRA5, PIM1, PIM2, MET, HTR3A, GRK5, CYP11B1, IMPDH2, PIK3CA, PIK3R1, F3, CYP19A1, BCAT2, TRPM8, ICAM1, SELE, MAOB, PIK3CD, PIK3CB, PIK3CG, MAPKAPK2, PARP10, CHEK2, CCNC, CDK8, MAPK10, AGPAT2, TGM2, SCD, XBP1, TYMS, DHFR, GABRA2, ROCK1, PRKACA, CHRM4, JAK2, LCK, CDK9, PTPN1, ALOX5AP, CHEK1, KIT, SRC, MKNK1, IKBKB, MAPK14, LRRK2, ROCK2, ATR, AOC3, GRK3, PLK1, GRK2, NPY5R, MAP4K4, NTRK1, ABL1, CDK1, CCNB1, NR3C2, PGR,

	DRD4, PTGS2, DRD3, CDK2, CDK4, SIRT2, ADORA2A, ADORA3, GRM5, QPCT, CSF1R, CNR2, CBFB, HSD17B1, PNMT, PARP2, PTGES, RPS27
Kaempferol	NOX4, AKR1B1, XDH, TYR, FLT3, CA2, ALOX5, CA7, HSD17B2, ABCC1, HSD17B1, AHR, CA12, ESRRA, ABCB1, CYP1B1, ABCG2, ADORA1, CA4, ACHE, MAOA, GLO1, SYK, GSK3B, MMP9, MMP2, ALOX15, ALOX12, PTPRS, ADORA2A, CDK5R1, CDK5, CCNB3, CDK1, CCNB1, CCNB2, ARG1, GPR35, ESR2, DAPK1, MPG, SLC22A12, TTR, AKR1B10, TNKS2, TNKS, CDK6, CDK2, CYP19A1, CSNK2A1, EGFR, AVPR2, IGF1R, F2, PIM1, AURKB, DRD4, MPO, PIK3R1, PYGL, CA1, SRC, PTK2, KDR, MMP13, MMP3, CA3, PLK1, CA6, PKN1, CA14, CA9, MET, NEK2, CXCR1, CAMK2B, ALK, AKT1, NEK6, PLA2G1B, CA5A, BACE1, AXL, NUAK1, AKR1C2, AKR1C1, AKR1C3, AKR1C4, CA13, AKR1A1, APP, PARP1, MMP12, CD38, TOP1, ESR1, PTGS2, CFTR, PFKFB3, AMY1A, GRK6, TERT, MAPT
Ligustilide	CA2, CA1, CTSK, SLC6A3, F2, PRSS1, CTSH, CTSL, CTSB, EPHX1, KCNK2, SLC6A4, ICAM1, VCAM1, SELE, CYP11B1, GABRA2, GABRB2, GABRG2, CYP11B2, ADRA2A, ADRA2C, ADRA2B, PDE10A, ELANE, TBXAS1, PIK3CD, PIK3R1, TRPM8, P2RX7, GPR55, ALDH2, PTGS1, PTGS2, CCR1, CCR5, CCR8, CHRM4, CHRM5, CHRM2, CHRM1, CHRM3, GABRB3, GABRG2, GABRA5, XPO1, FLT3, RAPGEF4, PGGT1B, FNTA, ADRA1D, CTRC, TGFBR1, MAOA, SLC6A2, ADRA1A
Linalool	TRPV3, CA2, CA1, CA4, TRPM8, NR3C2, NR3C1, PGR, SIGMAR1, SLC6A3, SQLE, IDO1, HSD17B2, DRD2, CHRM4, OPRM1, OPRD1, OPRK1, ADRA2C, HMOX1, JAK1, JAK2, PTGS2, KCNA5, PTAFR, SCN5A, SCN9A, PARP1, ADRA1A, HRH3, HRH4, JAK3, TYK2, TNNC1, TNNT2, TNNI3, LRRK2, AR
Loureirin A	ESR1, ESR2, ACHE, TUBB1, TTR, SIGMAR1, MMP2, MAOB, CHRNA7, HDAC8, MAOA, HSD17B2, RET, HSP90AA1, HDAC3, HDAC2, HDAC5, HDAC10, CHEK1, WEE1, ADORA2A, BMP1, MMP7, ADORA1, LNPEP, ADAM17, ABL1, MTOR, PIK3CD, PRKDC, PIK3CB, HCK, PIK3CG, PI4KB, PIK3CA, AURKA, EPHB4, PDE10A, CCND3, CCND1, CDK4, CCND2, ADAMTS4, MMP14, PTK2B, NCOR2, HDAC3, PFKFB3, HDAC11, ABCG2, ALOX5, CYP19A1, MIF, MAPKAPK2, HDAC4, HSD17B1, RAF1, PIM1, DYRK1A, PIM2, TLR9, MMP1, CCND1, CDK4, CCNE2, CDK2, CCNE1, JAK2, HTR2A, FLT3, OPRD1, PIM3, HDAC1, TBK1, HDAC7, DPP4, CTSS, HDAC9, AKR1B10, CYP24A1, CDK5R1, CDK5, FTO, PLK1, LIMK2, PIK3CA, PIK3R1, CASP3, SYK, ROCK2, CASP7, EP300, VCP, HPGD, PDGFRA, PDGFRB, FAAH, PDK1, BRAF, HSP90AB1, TNKS2, TNKS, LRRK2, GRK7, HIPK4, TAOK2, PIK3C2G, PIP4K2C, ERBB2, CSF1R
Loureirin B	ESR1, TUBB1, ACHE, ESR2, TTR, SIGMAR1, ALOX5, CHRNA7, ABCG2, WEE1, CHEK1, MMP2, CYP19A1, PDGFRA, PDGFRB, HSP90AA1, BMP1, RET, ADAM17, HDAC2, JAK3, CTSS, HDAC9, TLR9, LNPEP, MMP14, HTT, MIF, CYP1B1, PDE4B, ROCK2, MMP7, MAOB, PLK1, PDF, CDK5R1, CDK5, PIM1, TGFBR1, PIM2, HDAC3, NR1D1, TAS2R31, ABL1, PDE4A, HDAC5, PDE4C, HDAC11, HDAC4, NCOR2, HDAC3, MTOR, DPP4, PIK3CD, PRKDC, PIK3CB, HCK, PIK3CG, PI4KB, PIK3CA, HDAC10, EPHB4, MAPKAPK2, PIM3, FLT1, OPRD1, SYK, RAF1, CCND1, CDK4, CCNE2, CDK2, CCNE1, DYRK1A, BRAF, HDAC8, TNKS2, TNKS, MKNK1, AURKA, HDAC1, CFD, ADAMTS4, MAOA, BMP4, MMP25,

	FLT3, PDGFRA, MMP16, ADAM10, GSTP1, PDE10A, GSTM2, PIP4K2C, CDK2, CCNA1, CCNA2, SLC9A1, HSD17B3, GRM2, CSNK1D, PARP1, MCHR1, CLK1, CLK3, DYRK2, PRF1, HPGD
Loureirin C	ESR1, ESR2, ACHE, TTR, SIGMAR1, CYP19A1, ESRRA, ESRRB, DYRK1A, CLK1, WEE1, DYRK1B, MAOA, ADRA1D, INSR, HDAC5, HDAC7, ROCK1, HDAC4, PRKACA, RAF1, HSP90AA1, MMP2, ADAM17, EBP, TUBB1, HSD17B2, F3, NOX4, GCGR, BRAF, CHRNA7, DAO, MAOB, AURKB, AURKA, DNM1, HSD17B1, QDPR, CHEK1, HSD17B14, HDAC8, HDAC1, AKR1B10, MMP14, HDAC9, HDAC2, RPS6KA5, OPRD1, DUSP3, ABCG2, SGK1, RPS6KA3, CDK4, MT-CO2, PDF, TAAR1, YWHAG, FLT4, FLT3, PDGFRA, MMP16, LNPEP, DPP4, MAP2K2, CYP24A1, DCTPP1, CDK2, TYR, HDAC3, HDAC10, PDE5A, PDE7A, PIK3CG, RPS6KB1, RAC1, ANPEP, HSP90AB1
Luteolin	NOX4, AKR1B1, CDK5R1, CDK5, XDH, MAOA, FLT3, CA2, CCNB3, CDK1, CCNB1, CCNB2, ALOX5, ADORA1, CA7, GLO1, APP, SYK, GSK3B, PARP1, TTR, MMP9, CA12, MMP2, CA4, MMP12, CD38, CYP1B1, ABCG2, AKR1B10, TNKS2, TNKS, TOP1, ARG1, PTPRS, ABCC1, HSD17B1, ACHE, CDK6, ABCB1, HSD17B2, CYP19A1, ESR2, ADORA2A, CSNK2A1, ALOX15, ALOX12, ESR1, PTGS2, CFTR, AMY1A, GRK6, CA1, CA9, CDK2, TERT, CDK1, TYR, AHR, ESRRA, GPR35, AVPR2, IGF1R, EGFR, F2, PIM1, AURKB, DRD4, MPO, PIK3R1, DAPK1, PYGL, SRC, PTK2, KDR, MMP13, MMP3, CA3, PLK1, CA6, PKN1, CA14, MET, NEK2, CXCR1, CAMK2B, ALK, AKT1, NEK6, PLA2G1B, CA5A, BACE1, AXL, NUAK1, AKR1C2, AKR1C1, AKR1C3, AKR1C4, CA13, AKR1A1, PFKFB3, PLG, KDM4E, AR
Magnoflorine	DRD2, CHRNA4, CHRNB2, DRD3, DRD1, HTR1A, HTR7, HTR6, HTR2B, PTPRCAP, HTR2A, ADRA1D, TH, DRD4, HTR5A, ADRA1A, HTR1D, ADRB1, SLC6A3, DRD5, HTR2C, OPRM1, KCNH2, ADRA1B, TSPO, SLC6A4, HRH2, ADRA2A, ADRA2C, ADRA2B, HTR1B, PTGES, CDK5R1, CDK5, DYRK1A, MAPK14, RBP4, TRPC6, TRPC3, ALOX15, CYP19A1, IRAK4, ADRB2, JAK3, ERN1, STAT3, KDR, PIM1, MMP9, MMP2, MAPKAPK2, CDK1, PPARG, CCKBR, PPP5C, CCNB3, CDK1, CCNB1, CCNB2, GSK3B, NR3C2, EGFR, MGLL, ALOX12, AKR1B1, CASP3, MMP1, AURKA, SIGMAR1, SLC9A1, PREP, HSD17B3, RPS6KB1, FAP, RORC, CDK2, CCNA1, CCNA2, MAP2K1, AR, GABRA1, EPHX2, ADORA2A, GABRA5, MIF, HPGD, FLT3, MAPK3, MAPK1, CSF1R, PRKCG, LIPG, CCNE1, CDK2, HSD17B2, CCNE1, CDK3, PGR, DBF4, CDC7, ADAMTS5, PDGFRA, PDGFRB, SRC, MMP3, CYP17A1, FGFR1, EIF2AK2, F3, RPS6KA3, PDPK1
Marrubiagenin methyl ester	CYP19A1, PGR, ATP12A, AR, CYP17A1, CDC25A, CDC25C, HSD11B1, PTPN1, HSD17B2, CDC25B, NR3C2, TAS2R31, PPARG, AKR1C2, AKR1C1, NR3C1, PTGS2, HSD11B2, CES2, CYP2C19, HMGCR, PPP2CA, F2RL1, SHBG, AKR1C3, RPS6KA5, TTL, FNTA, FNTB, EPAS1, SRD5A2, IKBKB, PREP, HLCS, NOS2, CYP11B1, CYP11B2, ALOX5, PPP1CC, PTGES, CSF1R, LCK, MAPK14, KDR, JAK1, GSK3B, GRIK2, IL1B, ESR1, ESR2, EPHX1, SERPINA6, CTSK, CTSS, PDPK1, AKT1, PTPN2, AVPR2, MMP13, PDE7A, MMP1, PDGFRB, HRH3, HRH4, SMO, TBXAS1, HDAC3, HDAC8, HDAC11, HDAC10, FAAH, MTNR1B, PTAFR, PDE4D

Methyl gallate	FUT7, CA2, CA7, CA1, CA12, CA14, CA9, CA3, CA6, CA4, CA5B, CA5A, CA13, SQLE, SERPINE1, IGF1R, ALK, ESR2, TYR, BCL2L1, AURKB, SRC, PTK2, KDR, MET, NEK2, AXL, CNR2, TTR, POLA1, POLB
Nimbin	HSP90AA1, OPRK1, WNT3A, TNKS2, PARP1, F10, CHRM3, NR1H4, PDE10A, HSP90AB1, MDM2, PLAT, F2, MAPK14, ATR, CFD, CTSS, PSEN2, PSENEN, NCSTN, APH1A, PSEN1, APH1B, CNR1, CNR2, KIF11, MAP2K1, UTS2R, OXTR, CASP3, PYGL, CASP7, PDE9A, PDE1C, GCK, FLT3, CDK2, CCNA1, CCNA2, TACR1, JAK2, AVPR2, SMO, IDH1, KCNQ3, KCNQ2, PDE4B, PREP, BACE1, ADORA3, CYP2C9, NR1I2, CYP2C19, SYK, HDAC1, P2RX7, SCN9A, CPT1A, EPHX2, CPT2, MAOA, MAOB, KCNQ4, LRRK2, P2RX3, CALCRL, HCRTR2, GABRA5, HCRTR1, GPR119, MAPK8, PLA2G7, FASN, ALK, ROS1, FKBP1A, FKBP5, TDO2, AURKB, PFKFB3, SLC6A9, C5AR1, CAPN2, NTRK1, MMP3, SLC6A5, CDK1, MMP9, MMP1, CAPN1, IDO1, AURKA, BRD4, NAMPT, BRD3, JAK3, ADORA2B, JAK1, TYK2, CDK5R1, CDK5, EGFR, DRD2, HTR2A, DYRK1A, SEM1, PSMD8, PSMD4, PSMD13, PSMD7, PSMD6, PSMD11, PSMD12, PSMD3, PSMD2, PSMC5, PSMC3, PSMC6, PSMC4, PSMC1, PSMC2, ADRM1, PSMD14, PSMD1, PSMB7, PSMB4, PSMB3, PSMB11, PSMB10, PSMA8, PSMA7, PSMA6, PSMA5, PSMA4, PSMA3, PSMA2, PSMA1, PSMB6, PSMB9, PSMB8, PSMB5, PSMB1, PSMB2, PGGT1B, MAP3K8, TRPA1
Nimbolide	HSP90AA1, OPRK1, PDE10A, CHRM3, MDM2, CTSS, CTSV, CTSL, PSEN2, PSENEN, NCSTN, APH1A, PSEN1, APH1B, PDE9A, C5AR1, SCN9A, NR1H4, CNR1, ADORA3, CYP2C9, NR1I2, CYP2C19, UTS2R, MAPK1, TNKS2, F10, PDE1C, IDH1, CNR2, CDK5R1, CDK5, DRD2, HTR2A, DYRK1A, AOC3, CTSB, LRRK2, CDK2, CDK4, MAP2K1, RB1, CASP3, CASP7, PDE4B, BACE1, TAAR1, HTR2B, SIGMAR1, CCNA2, CDK2, HSD11B1, PTGER1, EGFR, EPHX2, NAMPT, CRHR1, FLT3, KCNH2, SRC, JAK2, P2RX3, TYK2, CALCRL, STAT3, HSP90AB1, PARP1, GCK, PFKFB3, HTR7, PDE1A, PDE1B, WNT3A, CCR1, NPY5R, LDHA, F2R, CDK2, CCNA1, CCNA2, JAK3, HTR2C, JAK1, ADORA1, IMPDH2, TDO2, ALOX5, CAPN2, BACE2, PYGL, CAPN1, IDO1, LIPG, LPL, PLAT, OXTR, F2, CA2, KCNQ3, KCNQ2, EPHB4, DUT, ERBB2, SCN5A, PIM1, MMP3, MMP9, MMP1, PIM2, PDE7A
N-trans-feruloyltyramine	MMP9, MMP2, MMP1, EGFR, CNR2, TYR, MAOB, PTGS2, BRAF, ALOX5, ALDH2, MMP3, MMP8, CHEK1, WEE1, DNM1, PRKCZ, THRA, CDK5R1, CDK5, THRB, CTSL, HSP90AA1, ABL1, SYK, GLI2, PDK1, BCHE, DRD2, DRD3, BMP1, CDK1, RPS6KB1, CDK1, CCNB1, CCNE1, CDK2, CCNE1, CDK3, GRK2, ACHE, EP300, DUSP3, ESRRA, ESRRB, HSD17B2, HSD17B1, INSR, EPHA2, EPHB2, EPHA5, EPHA4, EPHA8, EPHA7, EPHB3, EPHA3, EPHB1, EPHA1, MYLK, MTOR, TRPM8, MMP13, MMP7, AKT2, CDK4, MCL1, HPGDS, HDAC1, ANPEP, FNTA, FNTB, ADAM17, GRM2, HTR3A, AGTR1, ADORA1, ADORA2A, ROCK2, CA7, CA6, CA14, CA9, CA5A, VCP, ATP4B, ATP4A, CHEK2, PDE4D, PDE4C, MMP14, BRD4, TNF, BRD9, PNMT, BCL2L1, BCL2, SLC5A1, TRAP1, HSP90B1, TBXA2R, CFD, ALK, HSP90AB1, CA12, MELK, SPHK2

Obacunone	OPRK1, OPRM1, OPRD1, MIF, P2RX3, AOC3, BACE2, BACE1, CTSK, CTSS, CTSV, MAPK1, P2RX7, PSEN2, PSENEN, NCSTN, APH1A, PSEN1, APH1B, PDE7A, DRD2, HTR2A, PLA2G2A, PIM1, FBP1, PIM2, HMGCR, CDK5R1, CDK5, DYRK1A, HTR7, EPHX2, CTSL, PFKFB3, CCR1, CYP19A1, AVPR1A, C5AR1, ITK, CDK9, CDK2, CCNA1, CCNA2, PARP1, MDM2, SCN9A, EPHX1, OXTR, CFD, CNR1, SERPINA6, CNR2, MAPK14, HDAC1, SHBG, HDAC4, BRS3, LRRK2, MAOB, CCNE1, CDK2, SCN5A, NR1I2, CLK4, CLK1, CLK2, HSD11B1, AKR1C3, DYRK1B, PSMB8, SMYD2, PDE9A, F2R, CHRM2, CHRM1, CHRM3, SIGMAR1, MAP2K1, LIPE, NAMPT, ADRA2B, DRD3, ADORA3, KDR, CDK1, MET, FASN, TAAR1
O-Methylalloptaeroxylin	PDE4D, CYP1A1, CYP1A2, CYP1B1, CA7, CA1, CA12, CA9, ACHE, JAK2, MTNR1B, AR, TNFRSF1A, TRPA1, FLT3, PRKDC, ABCG2, RARA, IDO1, CA13, ADAMTS5, NOTUM, MAPK10, GABRB3, GABRG2, GABRA5, KCNA5, CCR8, MAPK14, TGFBR1, ACVR1B, CYP19A1, PARP1, NQO2, HSD11B1, PDE10A, HCRTR2, HCRTR1, CTSK, CTSS, CTSL, CYP11B1, CYP11B2, CYP17A1, P2RX7, SCN10A, CNR1, CNR2, DHODH, GAPDH, CCNC, CDK8, CCNE2, CDK2, CCNE1, GRM5, GSK3B, DYRK1B, BCHE, PDE3A, PDE7A, BDKRB2, PREP, HDAC3, HDAC6, JAK3, JAK1, HDAC1, TYK2, PARP2, MAPK8, PIM2, FAP, PDE2A, GUSB, ICAM1, NOS1, SELE, NOS2, HSP90AA1, GRM4, IKBKB, CCND3, CCND1, CDK4, CCND2, CDK9, CCNT1, CDK2, VCAM1, PIK3CA, HTR1A, DRD2, PDE4A, SRC, PDE4B, CHUK, PTGER2, TGM2, MAP2K1, BAZ2B, SYK, BAZ2A, MET, GABRA1, GABRB2, GABRG2, ADORA2B, CDK7, HTR7, HTR6
Palmatine	ACHE, HTR2B, BCHE, ADRA2C, ADRA2B, CHRM1, SIGMAR1, CYP2D6, SAE1, UBA2, RAC1, CDC42, MAP2K1, AURKB, AURKA, CDK2, CCNA1, CCNA2, PLK1, TTK, PLK3, PLK2, PGK1, TRPC6, MAPKAPK2, CCNC, CDK8, CDK8, HPGD, DYRK1A, GRM5, HTR3A, CYP1A1, NQO1, NQO2, CYP1B1, DRD4, PRF1, EPHX2, NTRK1, MTOR, MARK1, PIK3CD, PIK3CB, PIK3CG, KDM5B, ERBB2, GRM1, ABCG2, DHFR, CLK4, NAAA, PPIA, LCK, MET, LYN, EPHB4, TBXAS1, TEK, CDC25B, SCD, KIT, PIM1, CYP11B2, SLC1A3, CLK1, DYRK2, MDM2, CCND1, CDK4, GCK, ALDH2, MAPK8, MAPK10, MCHR1, HCRTR2, TRPV1, CSF1R, CFD, ROCK2, RAF1, IKBKB, HSD17B2, F3, FPR2, LRRK2, RPS6KA2, NAMPT, MST1R, CSNK1D, MMP3, CDK2, MMP9, CDK4, MMP1, SIRT2, FLT4, RET, ITK, HSD17B1, STAT3, HSD11B1, PTGER2, PDGFRB, PFKFB3
p-Coumaric acid	AKR1B1, CA2, CA7, ESR2, CA1, CA3, CA6, CA12, CA14, CA9, CA4, CA5B, CA5A, MIF, ALOX5, MMP9, MMP1, MMP2, PTPN1, AKR1B10, HCAR2, TLR4, CA13, F3, HSD11B1, TRPA1, ESR1, PGR, AKR1C3
Pectolinarigenin	KIT, OPRD1, AKR1B1, PIM1, ADORA1, ADORA2A, ADORA3, FLT3, CYP1B1, NOS2, NOX4, HSD17B1, ESR1, ESR2, ABCB1, ALOX15, ALOX12, ABCC1, APP, PTGS2, CFTR, GRK6, PTPRS, XDH, GSK3B, PLA2G2A, CA2, CA4, CDK1, CA9, HSD17B2, CYP19A1, MMP9, MMP2, OPRM1, CA7, CA12, CA1, CDK5R1 CDK5, MAOA, NAE1, AMY1A, ODC1, CCNB3 CDK1 CCNB1 CCNB2, CDK6, LCK, PIK3CG, ABCG2, ALOX5, KDM4E, CBR1, SIRT1, BACE1, TNKS2, TNKS, NTRK2, BCHE, CSNK2A1, ACHE, MCL1, TERT, EGFR, PLG, CALM1, MAOB, SYK, TTR, AKR1B10, ST6GAL1, CA6, AKT1, ALK, SLC22A12, SIGMAR1, AR, CYP1A1, CYP1A2, GLO1, ARG1, PFKFB3, IKBKB, MET, PARP1, MMP12, CD38, TOP1, HSP90B1, F2, AVPR2,

	CXCR1, GPR35, ALDH2, IGF1R, PPARG, PTPN1, TYR, AHR, ESRRA, HSP90AB1, AURKB
Perforatic acid	CYP1A1, CYP1A2, CYP1B1, PTGDR2, KDM5B, NR4A1, AKR1C3, PPARA, PPARD, PDE4D, SLC22A12, MKNK2, MKNK1, KDM4E, KDM3A, KDM2A, KDM5C, CNR2, METAP2, KDM4C, KDM2B, AKR1C2, CCKAR, SLC16A3, MAPK8, MAP3K8, PLEC, ITGB1 ITGA5, ITGB1 ITGA2, ITGB1 ITGA1, AKR1A1, CA1, ICAM1, SELE, SORT1, PTGER4, GRIK1, KMO, PLA2G2A, KDM4D, FABP4, ERCC5, FEN1, NR1H4, KDM6B, PRKDC, GYS1, CASP1, PIM1, ITGAV, DYRK2, PIM2, CAMKK1, CAMKK2, PIM3, TBXAS1, CDC25B, ECE1, P4HTM, TTR, MAPK1, CTSA, SCN10A, ACE, ITGAV ITGB3, MME, MMP2, PIK3CA, AURKA, PTGS1, ACE2, FFAR1, ADAMTS5, RHOA, HAO1, HSPA1A, CREBBP, CDC25A, MCL1, ALOX5AP, CDK2 CCNA1 CCNA2, CTSS, PLK1, ABL1, TDO2, IDO1, CPA1, CSNK2A1, ABAT, ITGB1 ITGA4, PFKFB3, PAM, PFKFB4, PFKFB4 PFKFB3, ERBB2, THRA, THRB, ITGAL ICAM1 ITGB2, PDE10A, BCL2
Perforatic acid methyl ester	MAPK8, KDM4E, KDM5C, KDM4A, KDM4D, KDM4C, CDK2 CCNA1 CCNA2, CDK2, PDE4D, PIM1, PIM2, MTNR1A, MTNR1B, NQO2, P2RX7, TAAR1, FLT3, CDK9 CCNT1, PIK3CA, ADAMTS5, CDK4, HSD11B1, FAP, CXCR2, ADORA2B, SYK, MAPK14, CSNK1D, GRM5, ADORA2A, PREP, PLCG2, JAK2, PDE10A, SRC, IKBKE, TBK1, HTR2C, LRRK2, KDM5B, CCNE2 CDK2 CCNE1, PDE4B, MET, IKBKB, ELANE, CA6, CA14, CA4, CTSS, SCN10A, TERT, CA7, CA12, CA9, CDC7, PIK3CD, PRKDC, PIK3CB, HCK, PIK3CG, PI4KB, EPHB4, FLT4, RET, RPS6KA1, PDE2A, PDE11A, KDR, MAP2K2, PDE7A, MAP2K1, FGR, RPS6KB1, TLR4, ABL1, CA2, AURKB, MTOR, MAPK9, JUN, CDK5R1 CDK5, HTR2A, GSK3B, GSK3A, IRAK4, PRMT3, RARA, CYP1A1, NOX4, TBXAS1, GRM4, KDM4B, KAT2B, PARP1, BCAT2, PARP2, CA1, PDGFRA PDGFRB, JAK3, MAPK10
Peucenin-7-methyl ester	NFKB1, PPARG, PDE4D, ALOX15, FASN, PTGS1, PTGS2, ALOX12, ABCB1, ABCG2, IDH1, MAOA, EGLN1, RELA, CNOT7, GRM5
Phloretin	ESR1, ESR2, ALOX5, ACHE, TYR, PLA2G5, NOX4, HSD17B2, HSD17B3, SHBG, HSD17B14, CDK4, PLA2G2A, YWHAG, CA6, CA5B, CA5A, SNCA, TERT, WEE1, NQO2, MAPK14, PGD, HSP90AA1, INSR, CDK5R1 CDK5, SFRP1, APP, PTGS2, IGFBP3, CDK1, HSD17B1, HDAC3, HDAC6, CCR5, CDK2, PTGS1, EIF2AK2, ERBB2, PLAT, CCNE2 CDK2 CCNE1, PLAU, MAOA, CHEK1, PDK1, MAOB
Physcion	ELANE, LIMK1, PTP4A3, CSNK2A1, LCK, ESR1, ESR2, PIM1, MCL1, DUSP3, EGFR, BCL2, FTO, EIF2AK2, MME, MMP3, MMP1, MMP9, MMP2, MMP8, PDE5A, NQO1, MMP13, CHRNA7, HDAC6, HDAC8, HDAC1, MMP7, MMP10, KDM1A, ADAM17, MAPK8, MMP16, MMP14, KCNMA1, MMP12, CTSV, BAD, TERT, MAP2K1, CYP19A1, CYP1B1, NOX4, BCHE, PLA2G7, GUSB, FLT3, NOTUM, LRRK2, HDAC5, HDAC7, HDAC4, HDAC9, ADAMTS5, ADAMTS4, PDE4B, PDE4C, MET, ANPEP, PIK3CD, CCNE1 CDK2, ERBB2, FLT1, PDGFRB, FLT4, PDGFRA, KDR, GRK6
Protocatechuic acid	CA2, CA7, CA1, CA6, CA12, CA14, CA9, CA4, CA3, CA5B, CA5A, CA13, FUT7, SQLE, LDHA, LDHB, TTR, ESR2, COMT, BCL2L1, IGF1R, ALK, SERPINE1, AKR1C3, GPR35, ALB

Pterostilbene	PTGS1, PTGS2, NQO2, CYP1B1, ESR1, AHR, TUBB3, TUBB1, RELA, CYP19A1, ABCB1, CA7, CA6, CA14, CA9, CA13, CA5B, CA5A, CYP1A1, CA2, APP, CA1, CA12, CA4, PIK3CA, CYP3A4, MAOA, CYP1A2, SLC6A2, CA3, PIK3CB, EGFR, LCK, MAPT, CYP2C9, CYP2C19, SYK, HMGCR, ALOX5, CDK5R1 CDK5, DYRK1A, MIF, ELANE, HSD11B1, FLT3, CHEK1, WEE1, HDAC3, HDAC1, BRD4, RAF1, GCGR, MAPK8, RPS6KA1, MAPK14, MAPK10, CSNK1D, PRKACB, MAP2K2, MAPK11, TTK, PRKACA, MAPK9, SLK, FRK, STK36, GAK, EPHA6, TNIK, PTK6, MAPK12, RPS6KA6, CSNK1E, RIPK2, CDC42BPB, DDR2, BRAF, ACVR1B, DDR1, NLK, ACVR2B, CSNK1A1L, CIT, CDC42BPG, COQ8B, LATS2, STK32B, JAK2, CYP17A1, CDK2, MMP3, PRF1, MCL1, BCL2, HDAC6, MTOR, MAPK1, HTT, ABL1, EPHX2
Quercetin	NOX4, AVPR2, AKR1B1, XDH, MAOA, IGF1R, FLT3, CYP19A1, EGFR, F2, CA2, PIM1, ALOX5, AURKB, DRD4, ADORA1, CA7, GLO1, MPO, PIK3R1, ADORA2A, DAPK1, PYGL, CA1, GSK3B, SRC, PTK2, HSD17B2, KDR, MMP13, MMP3, CA3, ALOX15, ABCC1, PLK1, CA6, CDK1, MMP9, CA12, MMP2, PKN1, CA14, CA9, CSNK2A1, ALOX12, MET, CA4, NEK2, CXCR1, CAMK2B, ALK, AKT1, ABCB1, NEK6, PLA2G1B, CA5A, BACE1, CYP1B1, AXL, ABCG2, NUAK1, AKR1C2, AKR1C1, AKR1C3, AKR1C4, CA13, AKR1A1, GPR35, MAPT, KDM4E, TOP2A, INSR, ACHE, MYLK, SYK, PIK3CG, APEX1, PTPRS, ESR2, MPG, SLC22A12, CDK5R1 CDK5, CCNB3 CDK1 CCNB1 CCNB2, ARG1, CDK6, CDK2, TYR, HSD17B1, AHR, ESRRA, APP, PARP1, TTR, MMP12, CD38, AKR1B10, TNKS2, TNKS, TOP1, TERT
Resveratrol	MAOA, CA2, ESR1, PTGS1, SLC6A2, PTGS2, CA7, APP, CA1, CA3, CA6, PIK3CB, CA12, CYP1A2, CYP2C9, CYP3A4, CA14, CA9, CYP2C19, CA4, CA13, NQO2, CA5B, PIK3CA, CA5A, LCK, SYK, CYP1B1, CYP19A1, ALOX5, TTR, AHR, TUBB1, EGFR, TUBB3, ABCB1, AKR1B1, CYP1A1, MMP9, MMP1, MMP2, RELA, TYR, CLK1, PTK2B, ESRRA, ESRRB, IGF1R, ALOX15, HSD17B2, HSD17B1, INSR, DYRK1A, DYRK1B, KIT, SRC, LTB4R, AR, ERN1, ESR2, HSD17B14, HDAC2, ABCC1, HDAC8, SHBG, CBR1, CYP11B1, CYP11B2, CYP17A1
Rhein	FTO, CYP19A1, ELANE, FNTA, FNTB, CSNK2A1, PTP4A3, ESR2, CASP3, PIM1, LDHA, LDHB, ERN1, ESR1, AMPD3, BCL2, ECE1, CDC25B, MCL1, LIMK1, GRK6, LCK, MME, CDK2, HNF4A, F2, IGFBP3, EGLN1, GPR35, SLC13A5, ACLY, CASP6, CASP7, CASP8, CASP1, CASP2, OGA, MAPK8, ADA, NOX4, CAMKK2, MMP16, MMP13, MMP9, MMP1, MMP2, MMP14, MMP8, INSR
Rhein methyl ester	FTO, ELANE, PTP4A3, FNTA, FNTB, CYP19A1, MMP9, MMP1, MMP2, MMP8, LDHA, LDHB, PRKCG, PRKCD, PRKCA, PRKCB, PRKCE, ESR2, PPARG, PIM1, PRKCH
Ralsolinol	DRD2, DRD3, DRD4, MAOA, PNMT, DRD1, SIGMAR1, MAOB, DHCR7, DRD5, ESR1, ESR2, TBXA2R, SLC6A2, SLC6A4, SLC6A3, ADRB2, HTR1A, ADRB1, ADRB3, HTR4, CHRNA4, HTR2A, ADRA2A, ADRA2C, PRCP, OPRM1, ABCC1, ABCB1, ANPEP, HTR1E, CHRM5, HTR2B, HTR2C, F3, ADRA1D, ADRA1B, QDPR, HTR3A, AADAT, OPRK1

Secoisolariciresinol	HTR1A, ALOX15, NR3C1, SHBG, TTR, ALOX12, IGF1R, PTGFR, POLA1, ALOX5, SRC, CYP24A1, MKNK2, CA2, CA1, GSK3B, CDK2, MAP2K1, WEE1, CHEK1, LYPLA1, LYPLA2, FLT3, PLK4, CDK5, AXL, STS, AR, KDR, ALK, CA4, NTRK1, SLC6A4, ADCY10, CA6, CALM1, MAP3K7, ESR1, RCOR1 KDM1A, MAP3K14, PYGL, CAPN1, SLC6A3, ESRRA, ESRRB, HMGCR, GPER1, P2RX7, ERN1, PIK3CD, PIK3CB, PIK3CG, MMP2, PIK3CA, CYP19A1, ESR2, MAP2K2, MAPKAPK5, TYK2, HSP90AA1, HSP90AB1, ADORA1, PRKCA, ADK, IRAK4, CNR2, MAP2K7, RELA, ITK, CSF1R, ACVRL1, GPR55, GPR18
Spathulenol	UGT2B7, HSD11B1, IDO1, PTGS1, NR1H3, PGR, SLC6A3, ICMT
Stigmasterol glucoside	IL2, STAT3, BCL2L1, PSEN2, PSENEN, NCSTN, APH1A, PSEN1, APH1B, PTAFR, PTPN1, PFKFB3, PTPN2, CDC25B, ACP1, F2, PPM1B, PPP1CC, PPP2CA, PPP2R5A, MET, HSD11B2, HSD11B1, S1PR3, S1PR1, DRD4, PPARA, GPR119
Syringaresinol	ALOX5, PTAFR, MAPK9, MCL1, SHBG, SLC5A2, SOAT1, HIF1A, SOAT2, SLC6A2, SLC6A4, CNR2
Tinocrisposide	SLC5A2, MAP2K1, OPRK1, SLC28A2, MAPK1, BRAF, SLC5A1, CTSD, ADK, MMP9, MMP2, MMP8, SLC29A1, CDK2 CCNA1 CCNA2, F3, LGALS3, LGALS1, SCN9A, ADORA1, ADORA2A, MME, ADORA3, SLC5A4, FOLH1, CA2, CA1, CA9, HCAR2, SLC5A11, ADORA2B, UPP1
Tinopanoid C	OPRK1, OPRM1, OPRD1, CTSS, ADAM17, CTSV, CTSL, CTSK, PSEN2, PSENEN, NCSTN, APH1A, PSEN1, APH1B, CNR1, MAPK14, MAPK11, TNKS2, PDE9A, WNT3A, CDK6, CDK2, CDK1, CDK4, PIM1, PIM2, SLC6A9, SLC6A5, DBF4, CDC7, PARP1, PDE1C, ALPL, P2RY12, DYRK1A, SIGMAR1, CLK4, CLK1, CLK2, DYRK1B, LRRK2, PTGER1, CASP3, CASP7, AURKA, IGF1R, C5AR1, AKR1C3, CDC7, CTSB, CHEK1, P2RX7, MAPK8, ESR2, MAPK10, EDNRB, CRHR1, JAK2, CDK5R1, CDK5, HMGCR, BACE1, MDM2, ERBB2, EGFR, TYMS, VDR, CA2, CA1, MAPK1, LTA4H, JAK3, GRM4, RHOA, BACE2, PDE4B, BRD4, CYP11B1, CYP11B2, CYP17A1, MAOA, CNR2, HTR2A, HTR2C, HRH1, NTRK1, CLK3, DYRK2, PDGFRB, TBXA2R, CHRNA7, KCNH2, CSNK1D, CSNK1E, LIPG, LPL, MAOB, SRC, CCNA2, CDK2, KCNA5, PDE10A, F10, GCK, GABRA5, TSPO, AOC3, KIF11
Tinopanoid D	OPRK1, CTSS, IMPDH2, AOC3, DRD2, C5AR1, BRD4, AVPR1A, BCHE, SLC27A1, LRRK2, CFD, CDK2, STK3, PSEN2, PSENEN, NCSTN, APH1A, PSEN1, APH1B, HTR2A, CTSK, BRS3, CYP11B1, CYP11B2, MTNR1A, MTNR1B, AGTR2, CASP3, PREP, CASP7, PIM1, PIM2, CTSV, CTSL, P2RX7, GRM5, STS, LIPE, GRM1, GSK3B, NR4A1, CLK4, ICMT, TSPO, NR3C1, PARP1, KCNA5, KCNA3, CASP1, STK26, RHOA, IDH1, PRKCG, TACR3, MAPK8, ADRA1A, MAPK10, NAAA, PDE10A, OXTR, JAK1, JAK2, MMP9, MMP1, GABRA5
Tinopanoid E	JAK3, JAK1, JAK2, TYK2, ROCK2, ROCK1, AR, PGR, CDC7, MMP1, MMP2, MMP8, F2RL3, CYP11B1, COMT, CYP11B2, CA1, CA14, CA4, CA13, EDNRA, PTPN1, PRKCA, BRD4, CREBBP, MAP3K14, PIM1, PARP1, PIM3, PLA2G1B, ADORA3, MAPK3, CYP19A1, ADORA2A, PYGL, MET, NPY5R, GSTM1, PRKDC, ADA, CA7, CA12, CA9, PRMT3, IARS, CA5B, KCNA5, CA5A, HSD11B1, TTL, CA2, MAPK14, CDK5R1, CDK5, PDE10A, ICMT, TOP2A

Tinopanoid F	OPRK1, ESR2, ESR1, JAK3, JAK1, JAK2, TYK2, IDO1, ATP12A, SHBG, NR3C1, LIPE, UGT2B7, TAAR1, PIM1, PIM3, CHRM4, NR3C2, CHRM5, CHRM2, CHRM1, CHRM3, CCNC, CDK8, CYP2C9, CYP2C19, CDK8, PER2, PDE10A, PSEN2, PSENEN, NCSTN, APH1A, PSEN1, APH1B, NPY5R, HSD11B2, PARP1, IL6ST, CHRNA3, CHRNB4, CCR1, SERPINA6, ADORA3, MAPK14, MAPK3, HSD17B2, DRD2, DRD4, NR1I2, MAP2K1, ALOX5, CNR2, AKR1C3, ABCC9, CA2, CA1, ROCK2, CA6, ROCK1, CA4, CDC42BPA, ICMT, LRRK2, GABRB3, GABRA3, GABRG2, GABRB3, GABRG2, GABRA1, GABRB3, GABRG2, GABRA5, GABRA2, GABRB3, GABRG2, CYP11B1, CYP11B2, PRMT3, GCGR, RPS6KA5, HSP90AA1, ST6GAL1, DRD3, FLT3, BRD4, MMP3, MMP9, MMP1, CREBBP, CCNB3, CDK1, CCNB1, CCNB2, CCR5, CDK1, PTGES, CYP17A1, HIF1A, MDM2, TRPA1, GABRA2, GABRB2, GABRG2, HSP90AB1, HMOX1, EGFR, SSTR3, EPHX2, P2RX7, CDK5R1, CDK5, DYRK1A, SIGMAR1, SRD5A1, SRD5A2, MTNR1A, GSK3B, F10, CDC7, MAOA
Tinopanoid G	F2RL3, CDC7, PIM1, PIM3, PRKCA, JAK1, PLA2G1B, AR, JAK3, TYK2, PTGS2, CYP11B1, CYP11B2, PGR, F2RL1, NPY5R, IARS, JAK2, PRKCD, COMT, MMP1, MMP2, MMP8, CYP51A1, ROCK2, ROCK1, TOP2A, CA2, PDE10A, CA7, CA3, CA6, CA12, CA9, CA5B, PCSK7, MAP3K14, BRD4, PTAFR, PRKDC, CREBBP, AOC3, CA5A, HMOX1, CDK5R1, CDK5, PYGL, POLA1, ADA, ADORA2A, MET, CHEK1, ATP2A1, MTNR1A, PRMT3, PRKCE, PARP1, GSTM1, LRRK2, MME, ACPP, MMP3, ADAM17, MMP14, CDC25A, MAPK14, XIAP, HSP90AB1, JAK3, JAK1, ICMT, PLA2G2A, ABCC9, ADORA3, MAPK3, KCNJ11, ABCC9, KCNA5, CYP19A1
Tinopanoid M	F2RL1, CDC25A, PTGS2, RPS6KA5, JAK1, PLA2G1B, CDC25B, ADA, JAK3, SYK, ZAP70, STAT5A, ATP1A1, CASP3, CASP6, CASP7, CASP1, PIM1, TTK, PIM2, PYGL, JAK2, TYK2, STAT3, MAPK14, MAPK11, LRRK2, GABRB3, GABRA3, GABRG2, GABRB3, GABRG2, GABRA1, GABRB3, GABRG2, GABRA5, CDK2, CCNA1, CCNA2, GABRA2, GABRB3, GABRG2, CXCR2, CAPN1, ICMT, HDAC6, HDAC1, PIM3, CYP11B1, HCRTR2, HCRTR1, MAP2K1, MET, CDK2, CDK9, PRKCA, F10, NPY5R, NLRP3, DPP4, ADAM17, CASP8, PIK3CA, PRMT3, COMT, IRAK4, CHEK1, F2RL3, KCNA3, ROCK2, ROCK1, NEK1, SCN9A
Tinopanoid R	OPRK1, OPRM1, AKR1C3, OPRD1, CCR1, NR3C1, PER2, HSD11B1, IMPDH1, IMPDH2, LRRK2, NR1I2, PDE10A, ALOX5, PSEN2, PSENEN, NCSTN, APH1A, PSEN1, APH1B, CNR2, BRD4, F10, CREBBP, CCNC, CDK8, CDK8, MAP2K1, MDM2, TERT, TTL, PYGL, PRKCB, MMP9, MET, CES2, DPP4, ADORA2A, NTRK1, HSP90AA1, PRKCG, JAK3, CDK6, CDK2, CDK1, CDK9, CDK4, CDK5, CDK3, CCNE1, CDK2, GABRB3, GABRA3, GABRG2, GABRB3, GABRG2, GABRA1, GABRB3, GABRG2, GABRA5, GABRA2, GABRB3, GABRG2, ITGAL, ICAM1, ITGB2, MMP1, MMP2, MMP8, NR3C2, CCNB3, CDK1, CCNB1, CCNB2, CDK2, CCNA1, CCNA2, RPS6KA3, SERPINA6, MAPK14, SHBG, CASP8, MAPK11, GYS1, CASP1, PAK1, CCND1, CDK4, CCNE2, CDK2, CCNE1, P2RX3, LIMK2, CSF1R, FKBP1A, MTOR, KDR, IRAK4, PDE5A, SLC5A2, FAAH, HSD17B2, CCKBR, MAPK1, TYRO3, MERTK
Tinospin C	OPRK1, OPRM1, AKR1C3, OPRD1, CCR1, NR3C1, PER2, HSD11B1, IMPDH1, IMPDH2, LRRK2, NR1I2, PDE10A, ALOX5, PSEN2, PSENEN, NCSTN, APH1A, PSEN1, APH1B, CNR2, BRD4, F10, CREBBP, CCNC,

	CDK8, CDK8, MAP2K1, MDM2, TERT, TTL, PYGL, PRKCB, MMP9, MET, CES2, DPP4, ADORA2A, NTRK1, HSP90AA1, PRKCG, JAK3, CDK6, CDK2, CDK1, CDK9, CDK4, CDK5, CDK3, CCNE1, CDK2
Tinosporol C	JUN, PGR, NR3C2, AR, PRKCA, PTPN1, PSEN2, PSENEN, NCSTN, APH1A, PSEN1, APH1B, IL1B, HMGCR, PARP1, NOS1, NOS3, NPY5R, TSPO, JAK3, JAK2, PPP2CA, CCND1, CDK4, GSK3B, HRH1, ADORA2A, ADORA2B, PARP3, PARP2, PARP4, TNKS, CCNB3, CDK1, CCNB1, CCNB2, PYGL, CAPN1, CMA1, CTSG, JAK1, CDK5R1, CDK5, MAPKAPK2, RPS6KA3, CYP11B1, EPHX2, HRH3, CYP11B2, AOC2, CYP19A1, NAAA, SLC6A9, ADORA3, TAAR1, TYK2, MGLL, ADRA2A, AKR1B1, ADRA2C, MPO, CTSS, ROCK2, ROCK1, FGFR1, HRH4, SCN9A, HNMT, MTNR1A, MTNR1B, AKT1, ABCG2, KCNK9, PTPRC, GPR139, P2RX7, CSF1R, CACNA1C, MKNK2, MKNK1, NAMPT, KCNH2, SYK, CTSK, MMP13, MMP1, CTSL, GRM2, CRHR1, PDE3A, PDE3B, HTR4, PIM1, TTK, PIM2, FAP, GABRA1, CCNE2, CDK2, CCNE1, CNR1, CSNK1G1, CSNK1A1, CSNK1D, NR1H2, CLK2, ADORA1, GABRB3, GABRG2, GABRA5, DRD4, DRD3, CCNC, CDK8, GRM5
Tinotufolin D	OPRK1, SLC6A4, OPRD1, OPRM1, SLC6A3, GABRA5, AKT1, CDK5R1, CDK5, MTNR1A, MTNR1B, CTSS, LRRK2, MGLL, MAPK8, MAPK14, MAPK10, SCN9A, PIK3CD, PIK3CB, CLK4, MAOA, KDR, PSEN2, PSENEN, NCSTN, APH1A, PSEN1, APH1B, PIK3CA, PIK3R1, MYLK, MTOR, PLK1, GRM5, PIK3CG, GRM1, PIK3CA, PLK3, EPHX2, NAAA, MALT1, MKNK1, ELANE, PRMT3, PDE9A, PDE10A, PDE1C, HPGDS, BRD3, MAOB, HTR1A, ADORA2B, CTSK, FPR1, FPR2
Tyramine	TAAR1, DRD2, DRD3, DRD1, DRD4, KDM4E, DRD5, MTNR1A, MTNR1B, SLC6A2, SLC6A3, SLC6A4, ADRA2A, ADRA2C, HTR3A, HTR6, HTR2A, GAPDH, NMUR2, AR, ESR1, ESR2, ESRRG, CHRNA7, ADRA2B, MIF, HTR2B, HTR2C, HTR1D, ADORA3, HRH1, SHBG, GABRA1, GABRB2, GABRG2
Vanillic acid	CA2, CA7, CA1, CA12, CA14, CA9, CA3, CA6, CA5A, CA4, TPMT, TTR, CA5B, CA13, FUT7, KDM4E, KDM4A, KDM3A, KDM6B, FTO, KDM4C, FYN, LCK, FBP1, AKR1C3, KDM2A, MMP9, MMP1, MMP2, MMP8, SQLE, POLA1, POLB, SERPINE1, TUBB1
ziganein	ELANE, PTP4A3, PIM1, ESR2, CSNK2A1, ESR1, FTO, MCL1, BCL2, FNTA, FNTB, CYP19A1, EGFR, LIMK1, LDHA, LCK, LDHB, DUSP3, HDAC6, HDAC8, HDAC1, CA7, CA14, CISD1, PLEC, CSNK1A1, CSNK1D, MMP13, MMP3, MMP1, HMGCR, NOX4, MMP16, MMP9, MMP2, MMP14, MMP12, MMP8, SRC, GRM5, EEF2K, RET, KDR, EIF2AK2, ABL1, MTOR, PIK3CD, PRKDC, PIK3CB, HCK, PIK3CG, PI4KB, PIK3CA, ASF1A, KCNMA1, ALPL, PLAA, HTR2B, GUSB, HDAC5, HDAC7, PLA2G7, HDAC4, ADAM17, ACE, ALPG, F2, CTSV, FLT1, FGFR1, NAT1, DAO
α-Santalol	UGT2B7, AR, SHBG, CDC25A, CDC25B, CYP19A1, IDO1, NR1I3, NPC1L1, RORA, ESR1, CHRM2, ACHE, SLC6A2, SLC6A4, CYP2C19, GCGR, PSEN2, PSENEN, NCSTN, APH1A, PSEN1, APH1B, KCNH2, PER2, PABPC1, ABHD6
β-Santalol	UGT2B7, SHBG, AR, CDC25A, CDC25B, IDO1, CYP19A1, GCGR, HSD17B3, KCNH2, PER2, NPC1L1, RORA, PABPC1, JAK1, JAK2, ABHD6, ESR1, CHRM2, ACHE, SLC6A2, SLC6A4, CYP2C19, PSEN2, PSENEN,

	NCSTN, APH1A, PSEN1, APH1B, SLC10A2, CHRM1, CHRM3, NR1I3, EPHX2, CHRM4, CHRM5, SLC18A3, CYP17A1, PIK3CG, PIK3CA

Table S3. The targets associated with PF.

PharmKrb-PF (97)	Genecards-PF (8,031)	OMIM-PF (11)
AHR, AKR1C1, ALOX15, ALOX5, AR, BMP1, BMP4, CTSG, CTSK, CTSL, CTSS, CYP27B1, EPHX1, F5, FGFR1, FPR1, GSTM1, HCAR2, HDAC1, HDAC10, HDAC2, HDAC3, HDAC4, HDAC6, HDAC9, HPGDS, IL1B, IL2, IL6, LTA4H, MAP2K4, MAP3K14, MAP3K8, MB, MIF, MPO, NFKB1, NOS2, NOX4, NR1D1, NR4A1, OXTR, P2RY12, PGR, PKN1, PLA2G2A, PLEC, PLG, PTAFR, PTGES, PTGES2, PTGS1, PTGS2, RARA, SELE, STAT3, STAT5A, TERT, THRA, TNF, TSPO, TTR, VEGFC, ADA, ADK, ADORA1, ADORA2A, ADORA2B, ADORA3, AKR1B10, CFD, CNR1, CNR2, DHFR2, MGLL, NAMPT, PDE10A, PDE11A, PDE1B, PDE2A, PDE3B, PDE4A, PDE4B, PDE4D, PDE5A,	ACCS, CNP, OAS1, OASL, HMGCL, HMGCR, HMGCS2, BDH2, HIBCH, KDSR, OXCT1, LOC107303338, LOC107303340, PAPSS2, PDPK1, ABAT, HOGA1, HPD, XRN1, ATIC, ALAS2, AZI2, HTR1A, HTR1D, HTR2A, HTR2C, HTR3A, HTR3B, HTR3C, HTR4, MTRR, NT5DC2, NT5E, NT5C1A, NT5C1B, NT5C3A, OPLAH, PFKFB1, PTS, OGG1, A2ML1-AS1, AHI1, ABHD16A, ABHD5, LOC107980440, ABI1, ABL1, LOC112679198, ABO, AIM2, LOC117134593, ACHE, ACAT1, ACAA2, ASMT, ASCL1, ACP1, ACP5, ASIC1, ANP32A, ACO2, ACOD1, ACR, ACRV1, ACTA2-AS1, ACTA1, ACTA2, ACTC1, ACTB, ABRA, ACTL9, ACTR3, ARPC1B, ARPC2, ACTN4, ATF6, ATF6B, AICDA, AHSA1, ACVRL1, ACVR1, ARC, APEH, ACBD3, ACAD8, ACADL, ACADM, ACADS, ACADSB, ACADVL, ACSF3, ACSL4, ACSM6, ACSS1, ACOT11, ACOT12, ACOT13, ACOT8, AGK, AOAH, ADAMDEC1, ADAM15, ADAM17, ADAM22, ADAM23, ADAM28, ADAM33, ADAM7, ADAMTS13, ADAMTS4, ADAM7-AS2, ADAM7-AS1, ADAMTSL1, AP3B1, AP1S3, AP5M1, AP3D1, AP4E1, ADD3, APRT, ADORA1, ADORA2A, ADORA2B, ADORA3, ADA2, LOC107303343, ADAR, ADARB1, ADARB2, ADAT3, ADK, AMPD1, AHCY, AMD1, ADCY9, ADCY10, ADCYAP1, AK2, ADSL, ADSS2, ADGRA2, ADGRE2, ADGRE5, ADGRL2, ADGRV1, APMAP, ADIPOR1, ADIPOQ, ARF1, ARFGEF1, ARL6, ARL11, ARL2BP, ARL5B, ARFRP1, ADPRH, ADRA1A, ADRA1D, ADRA2A, ADRA2B, ADRA2C, ADRB2, ADM, AGER, AVIL, AFDN, AFM, AFAP1-AS1, AFG3L2, AGMX2, AGBL3, ARMS2, ACAN, AGRN, AHNAK, AKAP3, AKAP14, AKAP17A, AKIP1, AKIRIN2, AKT1, AGXT, ANPEP, AARS1, AARS2, ALB, ADH1A, ADH1B, ADH1C, ADH4, ADH5, ADH7, ADHFE1, ALDH4A1, ALDH16A1, ALDH18A1, ALDH2, AOX1, AKR1A1, AKR1B1, AKR1B10, AKR1C1, ALDOA, AFF2, ALG1, ALG11, ALG12, ALG13, ALG14, ALG2, ALG3, ALG5, ALG6, ALG8, ALK, ACER3, ALPL, ALPG, ALPP, ALKBH1, ALKBH3, ALKBH5, AGMO, ALRH, AIF1, AIF1L, ALMS1, A4GALT, AHSG, AFP, GAA, AHSP, ALPK1, MGAT5, AMBP, A2M, A2ML1, FUCA1, IDUA, AMACR, NAGA, ALX3, ALYREF, AMBN, AMELX, AOC1, AOC4P, AIMP1, ACY1, AASS, ALAD, AMT, AMPH, AREG, AGL, APP, APBB1IP, APCS, ANAPC13, AR, ANG, AMOT, ANGPT2, ANGPTL6, ACE, AGTR1, AGT, ANLN, ANKH, ANK1, ANKFY1, ANKK1, ANKLE2, ANKMY1, ASB2, ANKS1A, ANKRD12, ANKRD17, ANKRD24, ANKRD27, ANKRD42, ANKRD46, ANKRD49, ANKRD55, ANKRD60, ANKRD61, ANKRD11, ANKRD26, ASZ1, ANXA1, ANXA10, ANXA11, ANXA2, ANXA4, ANXA5, ANXA6, ANXA8, ANO1, ANO10, ANOS1, AMN1, AMH, ATOX1, AIRN, AZIN1, ANTXR2, AAK1, AP4B1-AS1, APCDD1, AMER1, APC, APOC4-APOC2, APOA1, APOA4, APOE, APOBEC1, APOBEC3G, APOBEC3H, APOC1, APOC2, APOC3, APOL1, APOL2, APOL3, APOL4, AATF, AATK, AIFM1, API5, APAF1, APTX, APLF, APEX1, AQP5-AS1, AQP1, AQP10, AQP11, AQP12A, AQP2, AQP3, AQR, ALOX12B, ALOX12, ALOX15, ALOX5, ALOX5AP, BRAF, ARCN1, ACAP3, AGFG1, ARG1, RSRC1, AVP, AVPR1A, AVPR1B, AVPR2,	IL1B, IL2, IL6, NFKB1, NOS2, NOX4, PLA2G2A, PTGES, PTGES2, STAT3, TNF

PDE7A, PDE9A, PNP, SLC22A12, TPMT, TYMP, XDH, INSR, PRKCA, PRKCB, PRKCD, PRKCE, PRKCG, PTK2B, TRPA1, TRPM8, TRPV3, TYR, CPT2, FADS1, FASN, TKT	RERE, ASL, ASS1, RNPEP, ATE1, RARS1, RARS2, AGO2, AGO1, ARHGEF26-AS1, ARX, ARMC12, ARMC5, ARRB2, ARV1, AHR, AIP, AIPL1, AHRR, AADACL3, ARSD, ARSJ, ASGR1, ASPG, ASNS, NARS1, NARS2, ASPH, ASPDH, DRICH1, ASPRV1, ASPA, AGA, DARS2, ASPSCR1, ASRT3, ASTN2, ASXL1, LOC130058658, LOC130058885, LOC130058886, LOC130059053, LOC130059054, LOC129929080, LOC130059663, LOC130059892, LOC130059980, LOC130060043, LOC129929085, LOC130060959, LOC129929097, LOC129930930, LOC129929101, LOC129933155, LOC129934769, LOC129935044, LOC129935045, LOC129935046, LOC129935047, LOC129935405, LOC129936244, LOC129996027, LOC129929542, LOC130068093, LOC130068308, LOC130003807, LOC130006234, LOC130007232, LOC130007233, LOC130007242, LOC129930068, LOC129930245, LOC130063979, LOC130064021, LOC130064417, LOC129934333, LOC129935043, LOC129931648, LOC129936806, LOC129938041, LOC129992118, LOC129992304, LOC129997612, LOC129999940, LOC130001862, LOC130002910, LOC129929052, LOC130003418, LOC129929076, LOC129929077, LOC129929078, LOC130004025, LOC130004293, LOC129929079, LOC129929081, LOC130004578, LOC129929082, LOC129929083, LOC129929084, LOC129929086, LOC129929087, LOC129929088, LOC129929089, LOC129929090, LOC129929091, LOC129929092, LOC130006561, LOC129929093, LOC130006765, LOC129929094, LOC129929095, LOC129929096, LOC129929098, LOC129929099, LOC129929100, LOC129929102, LOC130008987, LOC129929103, LOC129929104, LOC129929105, LOC129929106, LOC130009810, LOC129929107, LOC129929108, LOC129929109, LOC129929110, LOC130055692, LOC129929111, LOC130055850, LOC130056217, LOC130058158, LOC130058479, LOC130058887, LOC130059760, LOC130059979, LOC130059981, LOC130060040, LOC130060041, LOC129930561, ATXN1, ATXN2L, ATXN10, ATCAY, ATL3, ATM, ATOH7, ABCB1, ABCA12, ABCC11, ABCB6, ABCC1, ABCC4, ABCG2, ATP5F1B, ATP5MC3, ATP5MK, ATPAF1, ATP2B1-AS1, ATP6V0E2-AS1, ATP13A1, ATP13A2, ATP7A, ATP7B, ATAD1, ATAD3C, ATP6AP1, ATP6V0A2, ATP6V0A4, ATP6V1A, ATP6V1B1, ATP6V1B2, ATP6V1E1, ATP6V1G2, ATP12A, ATP4A, ATP1A2, ATP1B1, ATP8B1, ATP2B4, ATP2A1, ATP2C2, ATRIP, ATR, ARID1A, ARID1B, ARID2, ARID3B, ARID4A, ARID4B, ARID5B, ATRIP-TREX1, ATN1, ATRX, ATXN8OS, ACKR1, ACKR2, AUH, AUP1, AURKA, AMFR, AIRE, ATG12, ATG16L1, ATG2A, ATG2B, ATG5, ATG9A, AXIN1, AXL, AXDND1, LOC106128902, LOC108004538, AZF1, AZU1, BTLA, BLNK, BCAP31, BANK1, B3GAT1-DT, BAALC, BABAM2-AS1, BPI, BIRC3, BCL7A, BCL7B, BCL7C, BANF1, BAG3, BAIAP2-DT, BAIAP2, BBS2, BSND, BPY2, BPY2B, BPY2C, BMAL1, BHLHE22, BZW1, BLZF1, BSG, BBX, BCAS2P2, BCL10, BCL11A, BAK1, BCL2, BAD, BAX, BBC3, BOK, BNIP3, BCL2L1, BCL2L10, BCL2L11, BCL2L12, BCL2A1, BCL3, BCOR, BCORL1, BCL6, BCL6B, BCL9, BCR, LOC107963955, LOC107963951, BCS1L, BDNF-AS, BFSP2, BECN1, BEST1, BGLT3, B4GALT1, B3GAT1, MGAT3, B2M, BCO1, LOC110006319, HBB-LCR, BHMT, BACE1, BTRC, UPB1, BID, BICC1, BICRAL, BLVRA, BLOC1S6, BTD, BIVM-ERCC5, BLK, BLM, BLOC1S5-TXNDC5, BMI1, BAMBI, BRINP3, BMS1P20, BMX, BOLA3, BRS3, BGLAP, BST2, BMP6, BMP10, BMPR1B, BMPR2,	

	BOLL, BPIFB1, BDKRB1, BDKRB2, BANCR, BASP1, BCYRN1, BDNF, BCAS1, BCKDHA, BCKDHB, BRAT1, BAP1, BARD1, BRCA1, BRIP1, BRCA2, BICRA, LOC126805577, LOC126805969, LOC126805994, LOC126805749, LOC126860933, LOC126860970, LOC126861339, LOC126861360, LOC126861451, LOC126861834, LOC126862860, LOC126862902, LOC126862841, LOC126806396, LOC126806658, LOC126806659, LOC126859963, LOC126860368, LOC126860369, LOC126863256, BCAR4, BLTP3A, BIN1, BROX, BAHD1, BAZ1A, BRWD1, BRD2, BRDT, BTK, BACH2, BUB1B, BTNL2, BTN2A1, BTN3A2, BCHE, BYSL, C1QTNF12, C2CD6, C9orf72, CA3-AS1, CBARP, CACNA1G-AS1, CDH1, CDH11, CDH13, CDH16, CDH17, CDH18, CDH26, CELSR1, CDH23, CDHR3, CALB1, CALCRL, CALCA, CALCB, CANT1, CIB3, CALCOCO2, CABP1, CACFD1, CASR, CACNA2D1, CACNB4, CACNG1, CACNA1A, CAMK2A, CAMK2D, CAMK2G, CAMK4, CASK, CACYBP, CALD1, CALM3, CAMTA1, CALML4, CANX, CAPN5, CAST, CALR, CASQ1, CREB1, CREB3L1, CREM, CAGE1, CASC11, CASC15, CASC8, CTAG1B, CT55, CT83, CT45A1, CT47A1, CT47A10, CT47A11, CT47A12, CT47A2, CT47A3, CT47A5, CT47A6, CT47A7, CT47A8, CT47A9, CT70, CT75, CNR1, CMTR1, CIC, CAPZB, CARMIL2, CPS1, CAD, CHST4, CA2, CA10, CA12, CA13, CA14, CA5A, CA5B, CBR1, CEL, CES1, CPA1, CPA2, CPA3, CPA6, CPB1, CPB2, CPQ, CPN1, CARD11-AS1, CARD8-AS1, CARMN, CRLS1, CRMA, CTF1, CLCF1, CPT1A, CPT2, CRTAP, CILP, COMP, CSN1S1, CSN3, CSNK2A1, CSNK1D, CSNK1E, CSNK1G2, CSNK2B, CLPX, CFLAR, CASP1, CASP10, CASP14, CASP16P, CARD11, CARD14, CARD9, CASTOR3P, CASZ1, CECR7, CAT, COMT, CTNNA1, CTNNB1, CTNNBIP1, CTNND1, CAMP, CTSB, CATSPER2, CDX2, CAVIN3, CAV2, CBFA2T3, CBL, CBLL1, CITED1, CBR3-AS1, CXCL8, CCL11, CXCL13, CCL17, CCL18, CCL19, CCL20, CCL21, CCL22, CCL24, CCL25, CCL26, CCL27, CCL3L1, CCR5, CCR10, CCRL2, CEBPA, CEBPB, CEBPD, CEBPE, CEBPZ, CTCF, CTCFL, CCDC169-SOHLH2, CCDC26, CCL15-CCL14, CCNO-DT, CNOT9, CCR5AS, CCZ1, CD101, CD14, CD163, CD164, CD177, CD180, CD19, CD1A, CD1B, CD1C, CD1D, CD1E, CD2AP, CD2-LCR, CD2, CD200, CD207, CD209, LOC117307477, CD22, CD226, CD24, CD244, CD247, CD248, CD27-AS1, CD27, CD274, CD276, CD28, CD3D, CD3E, CD300LD, CD300LD-AS1, CD320, CD33, CD34, CD36, CD37, CD38, CD4, CD40LG, CD40, CD44, CD46, CD47, CD48, CD5, CD5L, CD52, CD55, CD58, CD59, CD6, CD63, CD68, CD69, CD7, CD70, CD72, CD74, CD79A, CD79B, CD8A, CD8B, CD80, CD81-AS1, CD81, CD82, CD83, CD84, CD86, CD9, CD93, CD96, CD99, CD99L2, CDIN1, CDC42EP4, ARHGEF9, CDK5RAP1, CDK6-AS1, LOC126805874, LOC126805890, LOC126861013, LOC126860971, LOC126861452, LOC126861664, LOC126862123, LOC126862264, LOC126862361, LOC126862586, LOC126862865, LOC126806147, LOC126863160, LOC126860303, LOC126863253, LOC125467768, CIZ1, CDKN2B-AS1, CDIPT, CRPPA, CDR1-AS, CEACAM5, LOC110599576, CADM1, CAPRIN1, CIDEC, CDC14A, CDC14B, CDC25A, CDC25C, CDC27, CDC37, CDC40, CDC42, CDC6, CDC73, CCAR1, CCN2, CRABP1, CEMP1, CETN2, CENPB, CENATAC, CEP104, CEP120, CEP290, CEP70, CEP85L, CERS3, CERT1, CDR1, CRBN, CP, CCDST, CCEPR, CFTR, CFTR-AS1, LOC111674475, LOC113633877, LOC113664106, LOC111674477, LOC111674463, CCT7, CLC, CHMP2B, CHMP6, CHEK2, ENSG00000235775, CBY1, CHIT1, CHI3L1, CHIA, CHKB-CPT1B, CLCA1, CLIC1, CLCN2,	

	CLCNKA, CLCNKB, CHM, PSC, CCK, CCKAR, CH25H, CETP, CHKA, CHKB, CHAT, CHRM2, CHRNA1, CHRNB2, CHRND, CHRNE, CHRNG, CSGALNACT1, CSPG4, CNMD, CHRDL1, CGB3, CSH2, CHRFAM7A, CHAF1B, CDT1, CHTOP, CBX5, CHD2, CDY1, CDY1B, CDY2A, CHGA, C1orf141, C1orf159, C11orf65, C12orf75, C14orf132, DEL15Q11.2, DEL15Q13.3, C16orf92, C17orf107, DEL17Q12, C19orf12, C19orf53, C2orf69, C2orf88, C20orf204, C21orf91, C22orf39, C6orf118, C6orf132, C6orf47, C7orf33, LOC109504728, CSE1L, CMA1, CTRC, CTRL, CELA2A, CTRB1, CTRB2, CFAP410, CFAP43, CFAP45, CFAP47, CFAP52, CFAP54, CFAP61, CFAP91, CIMIP1, CNTF, CNTFR, CROCC, CPLANE1, CILK1, CATIP, CS, CLRN2, CLSPN, CIITA, CLTC, CLDN2, CLDN10, CLDN11, CLDN14, CLDN16, CLDN18, CLDN19, LOC106501713, CPSF4, CLN3, CLN5, CLN6, CLN8, CLOCK, CLPB, CLPTM1L, CLU, CNKSR3, F2R, F2, F3, F9, F5, F7, F8, F8A1, F12, F13B, CBLIF, COCH, CDAN1, COASY, COQ2, COQ4, COQ6, COQ8A, COQ8B, COQ9, CCHCR1, CC2D2A, CCDC40, CCDC102A, CCDC115, CCDC120, CCDC122, CCDC167, CCDC177, CCDC180, CCDC22, CCDC34, CCDC6, CCDC62, CCDC68, CCDC78, CCDC83, CCDC85A, CCDC85B, CCDC85C, CHCHD10, CHCHD5, COIL, CLPS, CCBE1, COLQ, COL1A1, COL2A1, COL3A1, COL4A3, COL6A5, COL7A1, COL11A2, COL13A1, COL17A1, COL18A1, COL20A1, COL25A1, COL26A1, COL27A1, COLEC10, CLTRN, CCAT1, CSF3, CSF3R, CSF2RA, CRNDE, COMMD3-BMI1, CERNA2, CERNA3, C1QB, C1QBP, C1R, C1S, C2, C3, C3AR1, CR1, CR2, C4A, C4B, C5, C5AR1, C6, C7, C8B, C9, C4BPA, C4B_2, CFH, CFHR1, CFP, CPLX1, CHUK, COG5, CRX, CNKSR2, CNTN1, CNTNAP2, COPS3, COPS7A, COPA, COPB2, COPE, COPG1, CPNE5, COMMD1, CPOX, CBFB, CIR1, CDB2, CDSN, CNIH1, CORO1A, CORO2A, CTTN, CTTNBP2, CRH, CRHBP, CRHR1, CORT, CPS1-IT1, CRP, CKB, CKMT1A, CKMT1B, CKMT2, CKM, CREBBP, LOC117038795, LOC115801415, CRKL, CRK, CRB2, CFC1, CRY1, CRYAB, CRYGC, LOC111188156, CSMD2-AS1, CTC1, CTAGE3P, CTAGE4, CTDP1, CSK, CTNS-AS1, CTPS1, CTR9, CTXN2-AS1, CLEC11A, CLEC16A, CLEC17A, CLEC5A, CLEC6A, CLEC7A, CLEC4M, CLEC12A, CLEC18A, CSMD1, CDCP1, CUBN, CELF2, CUL5, CUL4A, CUX1, CTAGE1, CX3CL1, CX3CR1, CXADR, CLMP, CXCL1P1, CXCL10, CXCL12, CXCL16, CXXC1, CAP1, CGAS, CNGB3, CCNA1, CCNA2, CNNM2, CCNB1, CCNB2, CCNB3, CCNK, DMTF1, CCND1, CCND2, CCND3, CCND3P1, CDK1, CDK11A, CDK11B, CDK13, CDK2AP1, CDKN1A, CDKN1B, CDKN1C, CDKN2A, CDKN2B, CDKN2C, CDKN3, CDKL5, CCNE1, CCNE2, CCNG1, CCNL2, CNTD1, CCNT1, CCNT2, CYLD-AS1, CYLD, LOC106799834, LOC110631417, LOC106780800, CBS, CST9L, CST3, CSRP1, CSRNP3, CHIC2, CRIP3, CSAD, CYSLTR1, CARS1, CTNS, CDA, CMPK1, CYBRD1, CYBA, CYBB, CYBC1, CYB5R3, CYB5A, COA3, COX18, COX20, COX10, COX15, COX4I1, COX4I2, COX5A, COX6A1, COX6A2, COX6B1, COX8A, CYCS, CYC1, CYP3A4, CYP11B2, CYP17A1, CYP19A1, CYP2A13, CYP2C18, CYP20A1, CYP21A1P, CYP21A2, CYP24A1, CYP26A1, CYP27B1, CYP4F22, CYP46A1, CYP51A1, POR, CYGB, CYTH1, CISH, CRLF1, CYFIP2, CLASP1, CKAP4, CYTOR, CIAO2B, CIAO3, CTU1, CTLA4, L2HGDH, DAB1, DACH2, DDB2, DAOA-AS1, DZIP1L, DBH-AS1, DCAF1, DCAF17, DDX1, DDX10, DDX17, DDX20, DDX23, DDX3X, DDX41, DDX54, DDX56, DEAF1, DEL15Q15.3, DHX36, DHX37, DHX8, DAPK1, DAXX, DBR1, DXO, PDSS2, DCN, DOCK8, DOCK10, DOCK11, DEF6, DEFA1, DEFB1, DEFB103A,	

	DEFB103B, DEFB104B, DEFB114, DEFB118, DEFB127, DEFB4A, DHDDS, DHTKD1, DHRS3, DEK, DAZ1, DAZL, DLEU2, DMBT1, DLL1, DLK1, DNER, DTX1, DMTN, DCANP1, DENND11, DENND1B, DENND2B, DTL, DMP1, DSPP, DCK, DGUOK, DHPS, DNASE1, DNASE1L1, DNASE2, DTYMK, DEPDC1B, DEPDC5, ATOD1, ATOD7, DPT, DDU, DES, DSC1, DSG2, DSP, DSTN, DEUP1, DPPA5, DDX60, DDX39A, DDX39B, DHX30, DHX58, DHX9, DGCR8, DIABLO, DGKA, DGKE, DICER1, DKKL1, DKK1, DANCR, DGCR11, DGCR5, DHFR, DBT, DLD, DLAT, DHODH, DPYSL5, DPYD, DUS2, DIP2C-AS1, DPEP1, DPP9, DPP10, DPP6, PPIP5K2, DPH3, DIS3, DIS3L2, DIP2B, DDR2, DLG1, DAAM2, DACT1, DVL1, CLLS2, DMAC1, DLX3, DLC1, DLEC1, DLGAP5, DMXL2, DCLRE1A, DCLRE1C, DDIAS, DDIT4, LIG4, DNMT1, DMAP1, DNMT3A, DNMT3B, DNTT, POLB, POLD1, POLD3, POLH, POLG2, POLG, POLI, POLK, POLL, POLM, POLN, POLQ, PRIM1, DNA2, TOP1, TOP2A, TOP2B, TOPBP1, TOP3B, DNAJA2, DNAJB1, DNAJB13, DNAJC12, DNAJC13, DNAJC14, DNAJC15, DNAJC19, DNAJC21, DNAJC24, DNAJC28, DNAJC3, DNAJC5, DNAJC6, LOC111674472, DND1, DALIR, DOK7, DOLK, DDOST, DPM1, DPM3, DPAGT1, DDC, DCT, DBH, DRD1, DRD2, DRD3, DRD4, DRGX, DOT1L, DUX4, DPF2, DMRT3, DRAIC, DR1, LOC117152611, LOC117152610, DPP9-AS1, DPY19L2, DPYD-AS1, DROSHA, DSCAM-AS1, DST-AS1, DTWD2, DUOX2, DUSP1, DUSP10, DUSP14, DUSP19, DUSP22, DUSP23, DUSP26, DUSP29, DYRK1A, DYRK1B, DYM, DCTN1, DNM1L, DNM2, DYNC2I1, DNAAF11, DNAAF2, DNAH8, DNAH10, DNAI1, DNAL1, DNALI1, DYNC1I2, DNHD1, DYNLL1, DYNLT1, DRC1, DBNDD1, DYSF, DKC1, DST, DTNB, DTNBP1, DAG1, DMD, EP300, E2F1, EMSLR, ELF4, EEA1, EGR1, EBF3, EBNA1BP2, EBP, EVI5, ECSIT, ENC1, EDA, EDAR, EDA2R, ENTPD2, ENPP1, EPG5, EDARADD, EDIL3-DT, EEF1E1-BLOC1S5, EFCAB10, EFCAB12, EFHC1, EFHD2, EFEMP2, EGFL8, EDIL3, EGFR-AS1, EGILA, EGLN1, ELAC2, ELANE, ELN, EMILIN2, ELAVL4, ETFDH, ETFA, ETFB, ERC1, ELOF1, ELL, EFL1, ELP1, ELOC, ELOVL4, EML4, EMB, ETDC, EMD, EMSY, EVL, ENAM, EBLN2, ERVW-1, ERVFRD-1, ERVK3-1, ENG, ENDOV, ERAP1, ERO1A, ERN1, ESM1, EDF1, EPAS1, EDN1, EDNRB, ELMO1, EDC4, EZH2, LOC106694316, ENO2, EVPL, EPX, EID2, EPB41L4A-DT, EPCIP-AS1, EPDR1, EPHA1, EPHA2, EPHA8, EPHB2, EPHB4, EFNA2, EFNA5, EFNB2, EFNB3, EGF, EGFR, EPS15, EPPIN, ECA1, GEFSP7, EREG, EPCAM, ECT2, EMP2, EPHX1, EDEM1, ERLIN2, EMC10, ERBIN, ERBB2, ERCC1, ERCC2, ERCC4, ERCC5, ERCC6L2, ERCC6, ERCC8, ERG28, ERMAP, EPB41, EPB41L1, ERFE, EPO, EPOR, ERAS, ESS2, EME1, ESCO2, ESR1, EBAG9, ESRRB, ETNK1, ETHE1, ETS1, ELK1, ERG, ETV6, ETS1-AS1, ERF, EHMT2, EEF2K, EEFSEC, EEF1A2, EEF1B2, EEF1G, EEF1GP1, EEF2, EIF6, EIF1AX, EIF2AK2, EIF2S1, EIF2S2, EIF2S3, EIF2B1, EIF2B2, EIF2B4, EIF2B5, EIF2B3, EIF3F, EIF4G1, EIF4E, EIF4EBP1, EIF5A, EIF5AL1, ETF1, EWSAT1, EWSR1, EXOC3-AS1, EXOC1, EXOC1L, EXO1, EXD1, EXOSC10, EXOSC5, EXT1, EXTL2P1, EXTL3, XPO1, ESPL1, EVR3, EYA4, EYS, EZHIP, EZR, F11R, F2RL3, F2RL1, FANCM, FANCD2, FAAP100, FAAP24, FIP1L1, FAM111A, FAM161A, FAM181A-AS1, FAM20A, FAM20C, FAM3B, FAM83A-AS1, FAM107A, FAM124A, FAM13A, FAM135A, FAM149B1, FAM163A, FAM167A, FAM168A, FAM170B, FAM177B, FAM184A, FAM216A, FAM225A, FAM30A, FAM53B, FAM76B, FAM81B, FAM83H, FAM89A, FANCD2OS, FUBP1, FDFT1, FAS-AS1, FAIM, FAF2, FADD, FAS, FASLG, FSCN1, FSCN2, FASTKD2, FAT1, FA2H, FAAH, FABP3, FABP12,	

	FABP5P3, FADS1, FASN, FAR1, FAU, FBXL4, FBXW11, FBXW7, FBH1, FBXO11, FBXO28, FBXO3, FBXO32, FBXO43, FBXL19-AS1, FCAR, FCER1A, FCER1G, FCER2, FCGRT, FCGR1A, FCGR2A, FCGR2B, FCGR2C, FCGR3A, FCGR3B, FCRL4, FCHO2, FEB1, FER, FER1L4, FRMD7, FARP1, FDX1, FRRS1L, FTH1, FTL, FTMT, FECH, FES, FATE1, FEZF1, FGD5-AS1, FGR, FHIP1B, FBL, FBN1, FGA, FGB, FGG, FGL2, FGF2, FGF10, FGF11, FGF12, FGF13, FGF14, FGF19, FGF21, FGF23, FGFBP2, FGFR1, FN1, FANK1, FBRS, FSIP1, FCN3, FIGN, FLG, FLNA, FBLIM1, FIBIN, FKBP10, FKBP11, FKBP1A, FKBP1B, FKBP5, FKBP6, FEN1, FLI1, FLII, FIZ1, FMR1NB, FLT3, FLT3LG, FOCAD, FALEC, FOLH1, FOLR1, FOLR2, FSHR, FDCSP, FL1, FLCN, FNIP1, FSTL1, FPGS, FOXA2, FOXG1, FOXI1, FOXI3, FOXJ1, FOXK2, FOXL2, FOXM1, FOXN1, FOXO1, FOXO3, FOXP1, FOXP2, FOXP3, FOXR2, FTCD, FMN1, FNBP1, FMNL2, FPR1, FOSL1, FOS, FHL1, FOXC2-AS1, FOXD2-AS1, FENDRR, FHIT, FMR1, FXN, FZD4, FRZB, FBP2, FRYL, FTO, FTX, FUOM, FUT10, FUT2, FUT9, FKRP, FH, FAH, FAHD2A, FURIN, FUS, FXYD2, FXYD6-FXYD2, FYN, FYCO1, GNL2, GPSM3, GNA11, GNA13, GNAI1, GNAQ, GNAO1, GNAT2, GNB1, GPBAR1, GPR12, GPR143, GPR15, GPR158, GPR18, GPR182, GPR32, GPR35, GPR55, GPR6, GPR65, GPR88, GPR89A, GPRC5A, GRK2, G3BP1, GABARAPL1, GABARAP, GALK1, GALNS, GALM, GALT, GLA, GLB1, GALC, GAL, LGALS3, LGALS3BP, GCASPC, GTSF1, GGN, GABRA1, GABRA2, GABRA3, GABRA5, GABRA6, GABRB1, GABRB2, GABRB3, GABRD, GABRG2, GABRG3, GABBR1, GGCX, GGH, GGACT, GGT1, GGT2P, GGTLC3, GM2A, GDAP1, GJA1, GJB2, GJC2, GAS5-AS1, GAS6-AS1, GSDMD, GHET1, GIP, GAST, GRP, GRPR, GATA2, GATA6-AS1, LOC106627981, LOC106627982, GC, GCN1, GDNF-AS1, GFRA1, GMDS, GMPPB, GSN, GEN1, GTF2B, GTF2E2, GTF2H1, GTF2I, GTF3C5, GPHN, GCNA, GCSAM, GFOD3P, GHRL, GAN, GIHCG, GIMD1, GIMAP1-GIMAP5, GINS1, GIPC2, GLI1, GDNF, GCM1, GFAP, GLIS1, GLMN, GCG, GLP1R, GCGR, GLCCI1, GCK, GCKR, GNE, G6PC3, G6PD, GPI, GANAB, GBA1, GBA3, GUSB, GAD2, GLUD1, GRIA2, GRID2, GRIK1, GRIN1, GRIN2A, GRIN2B, GRIN3A, GRM7, GLUL, GOT2, GPT, GLS, GATD3, GFPT1, QRSL1, QARS1, ENPEP, EPRS1, GATB, EARS2, GLRX5, GCDH, GPX4, GPX8, GSTM1, GSTP1, GSTT1, GSTZ1, GSS, GSR, GAPDH, GPD1, GATM, GLDC, GNMT, GLRA1, GLRA4, GLRB, PYGL, PYGM, GYS1, GSK3B, GYG1, GYPA, GYPC, GP2, GPA33, CGA, GP1BA, GPM6B, GPNMB, GP5, GP6, GPLD1, GLT1D1, GARS1, GLO1, GRHPR, GPC3, GNAS, GARIN5A, GOLM1, GOLPH3, GOSR1, GOLGA3, GOLGA5, GOLGA6L2, GOLGB1, GNRH1, GNRHR, GPANK1, GPKOW, GPATCH2L, GPATCH8, GPC3-AS1, GPN1, GRN, GNLY, GZMB, GAB2, GADD45A, GADD45B, GADD45G, GAS5, GAP43, GDF15, GFI1, GFI1B, GRB2, GFER, GH1, GHITM, GH-LCR, GHR, GHRH, GTF3C2-AS2, GUF1, GTPBP1, GTPBP3, GCH1, GAPVD1, GIMAP2, GAMT, GDA, GBP1, GUCY1A1, GUCY1B1, GUCY2D, GUK1, GET4, GUSBP1, H1-0, H1-10, H1-4, H19, HLX, H2AC4, H2AC11, H2AC12, H2AC13, H2AC14, H2AC15, H2AC16, H2AC17, H2AC18, H2AC19, H2AC20, H2AC21, H2AC25, H2AX, H2BC15, H2BC21, H2BW1, H3C1, H3C12, H3C13, H3-3B, H3-4, H3-7, LOC112533672, LOC112272621, LOC117125594, H4C5, H4C11, H4C12, H4C13, H4C14, H4C15, H4C16, HAND2-AS1, HP, HPR, HRK, HAS2-AS1, LOC106099062, HBS1L, HCGVIII-2, HCK, HAX1, HAND2, HSP90AA1, HSP90AA2P, HSP90B1, HSPA1L, HSPA12A, HSPA13, HSPA14, HSPA1A, HSPA1B, HSPA4, HSPB1, HSPD1, HSPE1, HSF1, HSFX1, HERC5,	

	HERC1, HACE1, HECW1, HHAT, HLTF, HELLS, HCLS1, HPGDS, HEBP1, HMOX1, HBA1, HBB, HBD, HBE1, HBG2, HBQ1, HBZ, HJV, HPX, HPLH1, HEPACAM2, HS6ST2, HSPG2, HS3ST3B1, HS3ST4, HGSNAT, HPSE, HBEGF, HDGF, HEPACAM, HAVCR2, HVBS7, HCCAT5, HEIH, HULC, HGF, HNF4A, HNF4G, HAMP, HEPH, HEPHL1, ENSG00000188078, HEYL, HNRNPA1, HNRNPA2B1, HNRNPK, HNRNPDL, HNRNPH1, HNRNPH2, HK2, HEXA, HEXB, H6PD, HHIPL2, HHLA2, HIC2, HIF1A-AS2, HIGD1A, HIGD1C, HMGA2, HMGB1, HMGN1, HPCA, HPCAL4, HNMT, HRH1, HRH2, HRH3, HRH4, HTN3, HDC, HRG, HINT1, HARS1, HARS2, HM13, HMSD, HDAC9, HDAC10, HTATIP2, HIVEP2, HCG25, HCG26, HCP5, HLA-DQB1-AS1, HLA-F-AS1, HLF, HNF1B, HNF1A-AS1, HNRNPK-AS1, HJURP, HLCS, HOXA10, HOXA13, HOXA2, HOXA5, HOXA7, HOXA9, HOXB9, HOXC13, HOXC4, HOXC5, HOXC6, HOXD13, HFE, HGD, HOOK1, HOPX, HORMAD1, HUNK, HRNR, HCFC1, HCFC2, HOTAIR, HOXA-AS2, HOTTIP, HOTAIRM1, HOXA11-AS, HAGLR, HPS1, LOC100500719, HPS3, HPS4, HPS5, HPS6, HRAS, ENSG00000266919, HSCB, HTR2A-AS1, HTRA1, HTT, HAP1, HIP1, HUS1, HAPLN1, HABP2, HMMR, HAS1, HYAL2, HYCC1, HAO1, HADH, HADHA, HADHB, HAGH, HCAR2, HSD3B2, HSD3BP4, HMBS, HSD11B2, HSD17B8, HSD17B10, HSD17B11, HSD17B13, HCN2, HCN1, HCRT, HCRTR2, HPRT1, HIF1A, HYOU1, ICAM4-AS1, IDS, IFI30, LOC119230225, IFNAR2-IL10RB, IFNG-AS1, IFT122P2, IRAIN, IGF2-AS, IGLON5, IK, IKZF1, LOC107988022, LOC107988021, LOC107988024, LOC107988025, IL12A-AS1, ITK, IL6-AS1, IER3, IER3IP1, IRGC, IRGM, IGBP1, IGES, IGHD, IGHE, IGHG1, IGHG2, IGHM, IGH, IGHV3-21, IGHV3-66, LOC102723407, IGKC, IGK, IGKV1D-33, IGLL1, IGSF3, IPO5, IDO1, ICOS, IVNS1ABP, INHA, INHBA, IBTK, ID2, ING1, IKBKG, IKBKB, IKBKE, INAVA, IMPDH2, ITPA, ITPR1, IMPA2, INPP5D, ITPKC, INS-IGF2, INSM1, INS, INSL6, IGF1, IGF1R, IGF2BP2, IGFBP6, INSR, INSRR, IRS4, INTS8, INTS10, INTS11, ITFG1, IBSP, ILK, ITGAM, ITGA2B, ITGB3, ITGBL1, IHO1, ITIH4, ICAM1, ICAM4, IFNA1, IFNA10, IFNA13, IFNA14, IFNA16, IFNA17, IFNA21, IFNAR1, IFI27, IFI6, IFNB1, IFNE, IFNG, IFI16, IFNGR1, IFI44, IFI44L, IFIT2, IFITM3, IFITM10, IFIH1, IFNK, IFNL3, IFNL4, IFNLR1, IFNW1, IRF3, IRF2BP2, ISG20, ISG20L2, IFN1@, IL1A, IL1B, IL1F10, IL1RAP, IL1RAPL2, IL1RN, IRAK4, IL1RL2, IL1R1, IL10, IL10RA, IL10RB, IL11, IL12RB1, IL12A, IL12B, IL13, IL13RA1, IL15, IL15RA, IL16, IL17RA, IL17A, IL17B, IL17C, IL17D, IL17F, IL18, IL18BP, IL18R1, IL18RAP, IL19, IL6, IL2RA, IL2RB, IL2RG, IL20RB, IL21, IL21R, IL22, IL22RA2, IL23R, IL23A, IL24, IL25, IL26, IL27, IL31, IL31RA, IL32, IL33, IL34, IL36A, IL36B, IL36G, IL36RN, IL37, IL4I1, IL7R, IL6ST, ILF2, ITSN1, ISX, IFT122, IFT56, IFT74, IFT81, INVS, INF2, IVL, IQANK1, IQSEC1, IQUB, IQCB1, IQCN, IQGAP3, IQCA1L, IREB2, ISCA2, IBA57, IRX2-DT, ISG15, ISL1, IAPP, IDH1, IARS1, IARS2, ICMT, IVD, IST1, ITCH, IATPR, ITPRID1, IZUMO4, JAG1, JAGN1, JAK2, JAZF1, JMJD7-PLA2G4B, JCHAIN, JPX, JRK, JRKL, JDP2, JUN, JUNB, JAM3, JSRP1, JPH1, KAZA1, KAZA2, KLK1, KLKB1, KLK10, KLK11, KLK14, KLK2, KPNA2, KPNB1, KANSL1, KCNQ1DN, KCNQ1OT1, KDELR3, KLHDC8B, KEAP1, KLHL7, KLHL24, KLHL31, KLHL33, KLHL40, KBTBD13, KBTBD4, KEL, KRT7, KRT10, KRT12, KRT14, KRT16, KRT18, KRT19, KRT20, KRT222, KRT24, KRT26, KRT37, KRT74, KRT76, KRT8P21, KRT81, KRT83, KRT85, KRT86, KRTAP9-2, KRTAP20-4, KDF1, KHDC1, KHDRBS3, KIAA0319, KIAA0319L, KIAA0586, KCP, KIR3DL1, KIR3DS1, KIR3DP1, KIR2DL3, KIR2DL5A, KIR2DL5B, KIR2DS2, KIR2DP1,	

	KLRC1, KLRC2, KLRC3, KLRC4, KLRD1, KLRG1, KLRK1, KLLN, KDR, KIF11, KIF13A, KIF1A, KIF1B, KIF21A, KIF21B, KIF5B, KIF6, KLC1, KNG1, KISS1R, KITLG, KIT, KLF1, KL, KLB, KMT2E-AS1, KRBOX4, KRBA1, KRAS, KREMEN1, KRTAP5-AS1, KTI12, KXD1, L1CAM, LARP1, LACC1, LALBA, LACTB, LDHC, LDHAL6A, LPO, LTF, LBX1, LAMA5-AS1, LMNA, LBR, LMNB1, LMNB2, LAMA3, LAMB3, LAMC2, LSS, LATS1, LARGE1, LCE2B, LCE3B, LCE3C, LAMTOR2, LCK, LRP1, LRP1B, LRAT, LCAT, MBL2, LGMN, LMOD1, LMLN, LEMD3, LPRS, LPRS6, LEP, LEPR, LEPROT, LEPQTL1, LAP3, LCMT2, LDC1P, LRG1, LGI1, LRMDA, LINGO1, LRRC17, LRRC18, LRRC25, LRRC32, LRRC41, LRRC4C, LRRC56, LRRC63, LRRC73, LRRC8A, LGR5, LRRK2, LGI2, LRIT1, LETM2, LZTR1, LNPEP, LARS1, LARS2, ALL2, ALL1, CLLS1, CLLS5, LUNAR1, LAIR1, LECT2, LILRA2, LILRA6, LILRB1, LILRB2, LILRB3, LTK, LST1, LTA4H, LTB4R, LTC4S, LIF, LIFR, LCORL, LIAT1, LNX2, LDB1, LIMD1, LPP, LMO1, LHX1, LMX1B, LIM2-AS1, LIN28B, LINS1, LAT, LIPA, LIPF, LIPJ, LIPI, LDAH, LPIN2, LCN2, LIAS, LSR, LBP, LPL, LPA, LLGL2, LMBRD1, LNCRNA-ATB, LNCBRM, LONP1, LINC01001, LINC01013, LINC01018, LINC01075, LINC01089, LINC01098, LINC01133, LINC01138, LINC01149, LINC01152, LINC01186, LINC01187, LINC01191, LINC01194, LINC01221, LINC01307, LINC01342, LINC01381, LINC01419, LINC01433, LINC01446, LINC01502, LINC01504, LINC01505, LINC01554, LINC01565, LINC01587, LINC01589, LINC00161, LINC01672, LINC00173, LINC01786, LINC01857, LINC01929, LINC01938, LINC02027, LINC00205, LINC02055, LINC02096, LINC00210, LINC02152, LINC02237, LINC02241, LINC02258, LINC00229, LINC02412, LINC02426, LINC02525, LINC02605, LINC00261, LINC02676, LINC02797, LINC00293, LINC02985, LINC03000, LINC03041, LINC00313, LINC00336, LINC00358, LINC00381, LINC00383, LINC00393, LINC00423, LINC00458, LINC00467, LINC00473, LINC00511, LINC00528, LINC00538, LINC00554, LINC00589, LINC00598, LINC00601, LINC00612, LINC00665, LINC00668, LINC00673, LINC00709, LINC00857, LINC00882, LINC00901, LINC00926, LINC00941, LINC00974, LINC-PINT, LINC-ROR, LORICRIN, LDLR, LDLRAD3, LRBA, LSM1, LSM11, LSM14A, LSM2, LSM4, LNPK, LCAL1, LUCAT1, LHB, LHCGR, LYAR, LY75-CD302, LYL1, LYVE1, LAG3, LY6E, LY6G5B, LY6G6D, LY75, LY9, LY96, LCP2, LSP1, LAX1, LEF1, LTA, LTB, LTBR, LYN, LYRM4, KAT2A, KAT2B, KAT6A, KAT6B, KAT8, KDM1A, KDM2B, KDM3A, KDM3B, KDM5A, KDM5C, KDM5D, KDM6A, KMT2A, KMT2B, KMT2C, KMT2D, KMT5B, LPCAT1, LAMP1, LAPTM4B, LYST, LYZ, LOX, LOXL2, KARS1, WM2, MAEA, MPEG1, MACIR, MIF, MSR1, MST1, MST1R, MROH6, MAF, MAFTRR, MAGEA1, MAGEA3, MAGEA4, MAGEA6, MAGEA9, MAGEA9B, MAGEB4, MAGEC2, MAGED2, MAGED4B, MAGEE1, MAGEL2, MAGT1, MPP2, MFSD11, MFSD12, MFSD14A, MFSD8, HLA-B, HLA-DMA, HLA-DMB, HLA-DOB, HLA-DPA1, HLA-DPB1, HLA-DQA1, HLA-DQB1, HLA-DRA, HLA-DRB1, HLA-DRB6, MIP, MVP, MKRN1, MDH2, ME2, MHS2, MALT1, MGAM, MGAM2, MPI, MRC1, M6PR, MPDU1, MAN1B1, MAN2A1, MANBA, MOGS, MAPKAPK3, MAPKAP1, MAPKAPK5-AS1, MROCKI, MKI67, MARVELD3, MRGPRX1, MRGPRX2, MILR1, MAML1, MEG3, MEG8, MATN1, MEPE, MGP, MMP1, MMP10, MMP11, MMP12, MMP13, MMP14, MMP19, MMP25, MMP26, MXRA8, MGA, MLX, MXI1, MASP2, MCL1, MCM3AP-AS1, MCTS1, MDM2, MDM4, MECOM, MTOR, LOC126805948, LOC126806063, LOC126862464, LOC126862662,	

	LOC126862707, LOC126862864, LOC126862866, LOC126807619, LOC126859807, LOC126859690, LOC126860438, LOC126863274, MED13L, MED17, MED19, MED20, MED25, MED27, MED29, MED8, MDC1, MEF2C-AS2, MEFV, MPIG6B, MATK, M1AP, MNS1, MEIG1, MEIS1, MLANA, MC1R, MRAP, MITF, MLPH, MELTF, MROS, MAGI2, MARCHF8, MBOAT7, MBTPS1, MME, MS4A1, MS4A2, MS4A4A, MEN1, MERTK, MIMT1, MEST, MSLN, MET, MMAB, MTDH, MPPE1, MT1DP, MT1E, MT1G, MT3, MTA1, MALAT1, MTX1, MTHFS, MAT1A, MAT2A, MSRA, METAP2, MARS1, MARS2, MBD4, MBD5, MECP2, MCCC2, MTHFD1, MTHFR, MCEE, MMUT, MSMO1, MTAP, METTL1, METTL13, METTL14, METTL16, METTL2A, METTL3, METTL5, METTL6, MVK, MFSD2A, MICA, MIA2, MIA-RAB4B, MIATNB, MICOS10-NBL1, MIR100, MIR101-1, MIR103A2, MIR106A, MIR106B, MIR107, MIR10A, MIR10B, MIR9-1, MIR1180, MIR1183, MIR1197, MIR1200, MIR1207, MIR122, MIR1233-1, MIR1238, MIR124-1, MIR1245B, MIR1246, MIR1247, MIR1248, MIR1251, MIR125A, MIR125B1, MIR126, MIR1262, MIR1263, MIR1264, MIR1268B, MIR127, MIR1275, MIR128-2, MIR1285-1, MIR1291, MIR129-1, MIR1293, MIR1299, MIR1304, MIR1307, MIR130A, MIR130B, MIR132, MIR133A1, MIR133B, MIR134, MIR135A1, MIR136, MIR137, MIR138-2, MIR139, MIR140, MIR141, MIR142, MIR143, MIR144, MIR145, MIR146A, MIR146B, MIR147A, MIR147B, MIR148A, MIR148B, MIR149, MIR150, MIR151A, MIR152, MIR154, MIR155, MIR15A, MIR15B, MIR16-1, MIR17, MIR181A1, MIR181B1, MIR181C, MIR181D, MIR182, MIR183, MIR184, MIR185, MIR186, MIR187, MIR188, MIR18A, MIR18B, MIR1908, MIR190A, MIR190B, MIR191, MIR1914, MIR192, MIR193A, MIR193B, MIR195, MIR196A2, MIR196B, MIR197, MIR198, MIR199A1, MIR199B, MIR19A, MIR19B1, MIR200A, MIR200B, MIR200C, MIR202, MIR203A, MIR204, MIR205, MIR2052, MIR206, MIR208A, MIR208B, MIR20A, MIR20B, MIR21, MIR210, MIR211, MIR2116, MIR212, MIR214, MIR215, MIR216A, MIR217, MIR218-2, MIR219A1, MIR22, MIR221, MIR222, MIR223, MIR224, MIR23A, MIR23B, MIR24-1, MIR25, MIR26A1, MIR26B, MIR27A, MIR27B, MIR28, MIR296, MIR299, MIR29A, MIR29B2, MIR29C, MIR301A, MIR301B, MIR302D, MIR3074, MIR30A, MIR30B, MIR30C1, MIR30D, MIR30E, MIR31, MIR3120, MIR3125, MIR3128, MIR3140, MIR3155A, MIR3158-1, MIR3173, MIR3175, MIR3178, MIR3180-1, MIR3183, MIR3184, MIR3187, MIR3199-1, MIR32, MIR320A, MIR323A, MIR324, MIR325, MIR326, MIR328, MIR329-1, MIR330, MIR331, MIR335, MIR337, MIR338, MIR339, MIR33A, MIR340, MIR342, MIR345, MIR34A, MIR34B, MIR34C, MIR3529, MIR361, MIR3614, MIR362, MIR363, MIR365A, MIR367, MIR3683, MIR369, MIR3691, MIR370, MIR371A, MIR372, MIR373, MIR374A, MIR374B, MIR375, MIR376A1, MIR376C, MIR377, MIR378A, MIR379, MIR381, MIR382, MIR383, MIR3909, MIR3937, MIR3940, MIR3944, MIR409, MIR412, MIR422A, MIR423, MIR424, MIR425, MIR4260, MIR4263, MIR4286, MIR4288, MIR429, MIR4291, MIR4299, MIR4304, MIR432, MIR4423, MIR4436B1, MIR4443, MIR4465, MIR4492, MIR449B, MIR451A, MIR452, MIR4520-1, MIR4523, MIR4536-1, MIR454, MIR455, MIR4659A, MIR4687, MIR4692, MIR4751, MIR4763, MIR4774, MIR4784, MIR4804, MIR483, MIR485, MIR486-1, MIR487A, MIR487B, MIR489, MIR490, MIR491, MIR493, MIR494, MIR495, MIR497, MIR498, MIR499A, MIR500A, MIR500B, MIR501, MIR502, MIR503, MIR504, MIR505, MIR508, MIR509-1, MIR510, MIR511, MIR512-1, MIR513A1, MIR513B, MIR515-1, MIR517A, MIR517B, MIR518A1, MIR518D, MIR518F, MIR519B, MIR520A,	

	MIR524, MIR525, MIR532, MIR542, MIR545, MIR548AA1, MIR548B, MIR548D1, MIR548G, MIR548H4, MIR548O2, MIR551B, MIR561, MIR562, MIR570, MIR5700, MIR574, MIR576, MIR582, MIR584, MIR587, MIR588, MIR589, MIR590, MIR596, MIR599, MIR605, MIR6069, MIR6080, MIR6086, MIR609, MIR6090, MIR6124, MIR6126, MIR615, MIR621, MIR625, MIR627, MIR628, MIR638, MIR641, MIR6499, MIR652, MIR653, MIR654, MIR655, MIR656, MIR660, MIR663A, MIR663B, MIR664A, MIR668, MIR671, MIR6726, MIR6727, MIR675, MIR676, MIR708, MIR744, MIR767, MIR7977, MIR873, MIR877, MIR885, MIR887, MIR888, MIR889, MIR92A1, MIR92B, MIR93, MIR95, MIR96, MIR98, MIR99A, MIR99B, MIRLET7A1, MIRLET7B, MIRLET7C, MIRLET7D, MIRLET7E, MIRLET7F1, MIRLET7G, MIRLET7I, MSMB, MGST1, MCRS1, MACF1, MARK2, MAP1LC3A, MAP1LC3B, MAP1LC3C, MAP1B, MAP2, MAPRE3, MAPT, MTUS1, MAST3, MAST4, MATCAP2, MDN1, MDK, MID2, MIF-AS1, MGR6, MFGE8, MINDY3, MCM10, MCM5, MSBP1, MIR100HG, MIR155HG, MIR17HG, MIR181A1HG, MIR210HG, MIR22HG, MIR223HG, MIR34AHG, MIR3945HG, MIR4435-2HG, MIR7-3HG, MIR99AHG, MSTO1, MINK1, MTARC2, MAVS, MCU, MTCH1, MICOS13, MIEF2, MTFR1, MPV17, MIPEP, MTFMT, MRPS12, MRPS22, MRPS30, MRPS7, MRM2, MTERF1, MTRF1, MTIF2, MTO1, MT-RNR1, MT-RNR2, MT-ATP6, MT-CYB, MT-CO1, MT-CO2, MT-CO3, MT-ND1, MT-ND4L, MT-TA, MT-TR, MT-TN, MT-TD, MT-TC, MT-TQ, MT-TE, MT-TG, MT-TH, MT-TI, MT-TL2, MT-TL1, MT-TK, MT-TM, MT-TF, MT-TP, MT-TS2, MT-TS1, MT-TT, MT-TW, MT-TY, MT-TV, MFN2, MAPK1, MAPK1IP1L, MAPK10, MAPK14, MAPK8IP3, MAP2K2, MAP3K8, MAP3K14, MAP3K20, MAP4K4, MALINC1, MAD2L2, MZT2A, MKKS, MKRN2OS, MKS1, MLLT1, MLLT10, MLLT11, MLLT3, MLXIPL, MME-AS1, MMP2-AS1, MMS19, MN1, MLC1, MSN, MKX, MOCS1, MON1A, MAOB, MGLL, MORC3, MORN1, MOSPD3, MLN, MOV10L1, MOV10, MPHOSPH9, MPLKIP, MPL, MPND, LOC129391106, LOC129388938, LOC129388419, LOC129389544, LOC129388420, LOC129388421, MPV17L, MSX2, MSL2, MTCO3P1, MTSS1, MUC1, MUC12, MUC16, MUC2, MUC20, MUC3A, MUC5AC, MUC5B, MUC7, MCOLN1, MADCAM1, MFHAS1, MPDZ, MS, MS2, MS4, MUS81, MSI2, MUSK, MRAS, MLH1, MSH5, MUTYH, MVP-DT, MX1, MYB, MAX, MAZ, MYC, MINCR, MYCL, MYCNOS, MYCN, MYCNUT, MTBS1, MYD88, MAG, MOBP, MBP, MOG, MPZ, MYT1L, MDS2, MNDA, MLF1, MYEOV, MPO, MYG1, MIAT, MRTFA, MYOC, MEF2A, MEF2B, MDFI, MDFIC, MYOD1, MYOG, MB, MYOM2, MYBPC3, MYBPH, MYH7, MYH11, MYH14, MYHAS, MYO1C, MYO1E, MYO9A, MYO9B, MYL2, MYL6B, MYLK, MYO5A, MYO5B, MYO7A, MYRIP, MYO19, MYO18B, MSTN, MYOT, MTPN, MTM1, MTMR7, MYOZ2, NAGLU, GNPTG, GNPTAB, NAGPA, NAGS, NANP, NAT2, NAT9, ASAH1, NQO1, NAXD, NAXE, NDUFAF2, NDUFS1, NDUFS2, NDUFS7, NDUFS8, NDUFV1, NDUFV2, NDUFA1, NDUFA11, NDUFA2, NDUFA5, NDUFA6, NDUFA9, NDUFB11, NDUFB4, NDUFB8, NDUFB9, NDUFC2, NDUFS4, NDOR1, NOX1, NOXA1, NOXO1, NAA10, NANOG, NANOS1, NAPSA, NARF-AS1, NACA, NPPB, NPR1, NCR3, NKG7, NAXD-AS1, NBAS, NBL1, NBPF3, NBPF10, NBPF11, NBPF12, NBPF13P, NBPF14, NBPF15, NBPF17P, NBPF18P, NBPF19, NBPF7P, NBPF20, NBPF21P, NBPF22P, NBPF25P, NBPF26, NBR1, NCF4-AS1, NCK1, NCKAP1L, NCKIPSD, NCK1-DT, NDST1, NDP-AS1, NDUFV2-AS1, LOC132090450, NEB, NECTIN4, NEDD4, NELFA, NRAV, NRIR, NEIL1, NLK, NPHP4, NGF, NGFR, NHLH2,	

	NES, NSF, NTNG1, NTNG2, NCAM1, NEU1, NRG1, NEXMIF, NBEAL2, NBEAP1, NBAT1, NHEG1, NBLST4, NFASC, NF1, NEFH, NEFL, NEUROG2, NLGN2, NMB, NMBR, NAV1, NRCAM, NEUROD2, NEGR1, NPAS2, NPTX2, NPTXR, NREP, NPFF, NPSR1, NPY, NPY5R, NRP2, NETO1, NPTN, NTS, NTRK1, NTF3, NCF1, NM, NEXN-AS1, NF2, NFE2L2, NFIA-AS2, NKILA, NFKBIA, NFKBIB, NFKBIE, NFKBIL1, NKRF, NFU1, NGF-AS1, NAB2, NGLY1, NHERF1, NHLRC1, NHS, NBN, NNMT, NMNAT1, NAMPT, NAMPTP2, NEK7, NINL, NIPAL4, NIPA2, NIPBL, NISCH, NOS2, NKX2-1, NKX2-1-AS1, NAIP, NLRC4, NLRP3, NLRP11, NLRP12, NLRP13, NME2, MPG, NMRAL1, NMRAL2P, NMT2, NNT-AS1, NOG, NORAD, NCMAP, NHEJ1, NONO, NCAPG, NCAPH2, NSUN2, NOP53, NDP, LOC111365141, NOTCH2NLA, NOTCH4, NALT1, NOVA1, ENSG00000228778, ENSG00000266708, ENSG00000273486, ENSG00000265413, NPC1, NPHP3-AS1, NPHS1, NPHS2, NPRL2, NPRL3, NPSR1-AS1, NPTN-IT1, NR2F1-AS1, NRAS, NQO2, NSA2, NSMCE3, NAPA, SGSH, NT5C1B-RDH14, NTAN1, NTHL1, NUAK2, NUBPL, NFIX, NFKB1, NFAT5, NFE2, NUMA1, NEAT1, NPIPB8, NPAT, NRBP1, NSD1, NCOA3, NCOR2, NR1I2, NXF1, NFYA, NFYB, NFYC, NXT1, NABP1, NUCB1, NCL, NPM1, NPM3, NUP107, NUP133, NUP160, NUP205, NUP210, NUP214, NUP37, NUP85, NUP93, NUP98, LOC106865369, NTPCR, NAP1L4, NOD2, NUDT10, NUDT16, NUDT6, LOC107126288, LOC107197952, LOC107133509, NUTM1, NYX, MGMT, OARD1, OBSCN, OCA2, OCLN, OCRL, ODAPH, ODAM, OBP2A, OFD1, OGA, OLAH, OLFM1, OMP, OR1F1, OR1F2P, OR10G6, OR2AK2, OR4C16, OR51I1, OR8K3, OLIG2, OPALIN, OCM, OSM, OSMR, ONECUT1, OPA1, OPRD1, OPRM1, OPN1LW, OPN4, OPTN, ORAI1, LOC107133510, LOC107181288, ORC1, ORMDL3, OAT, ODC1, OAZ3, OTC, OFCC1, ORM1, OSGEP, OSGEPL1, OSTF1, OGN, OMS, OTOF, OTOG, OTUD5, OTUD7A, OTULIN, OTULINL, ODF1, ODAD2, OPA3, OVCH1-AS1, OXA1L, OXSR1, OXER1, OXGR1, OSBP2, OSBPL1A, OSBPL7, OXTR, OXT, P2RY8, LOC126805576, LOC126862230, LOC126806757, PRECSIT, PRAL, PAF1, PAX5, PILRA, PHOX2A, PHOX2B, PITX2, PALLD, PALMD, PPT1, PTF1A, PDX1, PNLIP, PANX1, PANK2, PAPPA-AS1, PAPPA, PON1, PTH, PTH1R, PTHLH, PACRG, PACRGL, PRKN, PARK7, PNLDC1, PALB2, PVALB, PASD1, PATL1, PNPLA3, PTCH1, PTCHD4, PEG10, PAUPAR, PAX8-AS1, PXN, PBX2, PSIP1, PCBP2-OT1, PCNA-AS1, PCLAF, PLUT, PDLIM7, PBK, PDZK1, PDZK1IP1, PELI1, PTX3, PEPD, PI15, PI3, PMPCA, PDF, PYY, PGLYRP1, PADI4, PIN1, PPIC, PTRH2, PTRHD1, PRF1, PCM1, PLIN1, PER2, POSTN, PMP2, PMP22, PRPH2, PPL, PXDN, PXD	

Table S4. Information in topology parameters of intersection targets.

Intersection targets	Degree	Betweenness Centrality	Closeness Centrality
TNF	69	0.246112565	0.658031088
IL6	54	0.099402268	0.604761905
IL1B	54	0.100553607	0.604761905
PTGS2	45	0.086126476	0.561946903
STAT3	40	0.055452192	0.531380753
NFKB1	37	0.036232902	0.516260163
HDAC1	25	0.038500207	0.484732824
PRKCA	22	0.028541617	0.46350365
MPO	22	0.012274696	0.482889734
PTGS1	19	0.009810544	0.455197133
AR	19	0.025798136	0.473880597
ADK	19	0.035888295	0.399371069
XDH	19	0.024907998	0.465201465
IL2	19	0.017711619	0.479245283
HDAC2	18	0.016181226	0.453571429
MAOA	18	0.024909963	0.468634686
PLG	17	0.050450195	0.455197133
MAOB	17	0.023453383	0.472118959
PGR	16	0.012386377	0.461818182
ALOX5	16	0.013169939	0.442508711
ADA	16	0.02052131	0.466911765
TSPO	15	0.029652531	0.455197133
ADORA2A	15	0.033939387	0.465201465
CTSS	15	0.002688183	0.455197133
STAT5A	14	0.002508907	0.442508711
SELE	14	0.007454777	0.451957295
HDAC4	14	0.005228334	0.448763251
BCHE	14	0.00118322	0.447183099
HTT	13	0.014550043	0.458483755
TERT	13	0.002199569	0.439446367
FASN	13	0.048124023	0.456834532
HDAC3	13	0.004471089	0.403174603
NOS2	12	0.013057689	0.447183099

PRKCD	12	0.00555956	0.433447099
TTR	12	0.006288823	0.440972222
BACE1	12	0.00411996	0.442508711
INSR	11	0.005648135	0.405750799
RARA	11	0.002674648	0.396875
CTSG	11	0.001902581	0.427609428
NAMPT	11	0.008361588	0.436426117
PSEN1	11	0.0075329	0.436426117
PRKCB	11	0.011870288	0.436426117
HDAC6	11	0.00131807	0.437931034
PTGES	11	0.001073629	0.427609428
FPR1	11	0.003602475	0.429054054
PDE4A	11	0.050937786	0.439446367
PDE10A	11	0.014433454	0.368115942
NOX4	10	3.01E-04	0.434931507
NR4A1	10	0.001496978	0.437931034
CTSK	10	6.63E-04	0.424749164
TRPA1	10	0.027513669	0.429054054
CYSLTR1	10	0.025401596	0.419141914
LTA4H	10	0.001788007	0.430508475
P2RY12	10	0.007269635	0.434931507
PDE11A	10	0.002780845	0.339572193
MIF	9	0.002868898	0.434931507
TOP2A	9	0.04855026	0.381381381
PLA2G2A	9	2.95E-05	0.420529801
ALOX15	9	2.95E-05	0.420529801
FGFR1	9	0.002107863	0.409677419
PDE4D	9	0.001320534	0.345108696
PDE2A	9	0.001603591	0.335092348
PDE1B	9	0.001603591	0.335092348
PDE5A	8	0.005458372	0.331592689
PRKCE	8	0.005159545	0.375739645
HDAC9	8	4.33E-04	0.367052023
VEGFC	8	0.001516444	0.431972789

PTGES2	8	0.004431137	0.424749164
PTAFR	8	0.001878834	0.427609428
TYR	8	0.00116217	0.434931507
PDE7A	8	2.48E-04	0.334210526
DHFR2	8	0.016386965	0.416393443
MAP3K8	7	4.78E-04	0.41503268
MB	7	0.010397416	0.423333333
HPGDS	7	0.002547998	0.389570552
CTSL	7	0.003043328	0.409677419
EPHX1	7	0.00853624	0.354748603
CNR1	7	0.005299191	0.395638629
BMP4	7	0.004956905	0.426174497
TPMT	7	0.007062298	0.375739645
PNP	7	0.002824221	0.401898734
GLO1	6	0.007846029	0.355742297
MGLL	6	0.0053912	0.391975309
AKR1B10	6	0.007305726	0.361823362
PDE4B	6	0.008585373	0.448763251
ADORA3	6	0.003128984	0.369186047
PTK2B	5	4.90E-04	0.419141914
PDE9A	5	0	0.279735683
PRKCG	5	0	0.338666667
THRA	5	3.97E-04	0.351800554
HDAC10	5	0	0.331592689
GSTM1	5	0.003379753	0.416393443
OXTR	5	0.001289359	0.41503268
CYP27B1	5	7.17E-05	0.416393443
HCAR2	5	0.004381642	0.409677419
TBXA2R	5	7.69E-04	0.354748603
GCGR	5	0.010384202	0.412337662
TKT	5	0.006694386	0.376854599
MAP2K4	4	7.34E-05	0.416393443
HAO1	4	0.016284701	0.326478149
TRPM8	4	8.05E-04	0.321518987

CNR2	4	4.92E-04	0.333333333
TYMP	4	2.32E-04	0.357746479
PDE3B	4	0.002993245	0.347945205
ADORA1	4	1.14E-04	0.348901099
ADORA2B	4	0.001704386	0.349862259
TDP1	3	0.015748031	0.278508772
MAP3K14	3	0	0.401898734
NR1D1	3	1.15E-04	0.336870027
F5	3	0.001181171	0.330729167
CFD	3	0	0.404458599
BMP1	3	2.20E-04	0.323155216
AKR1C1	3	9.42E-04	0.287981859
TRPV3	2	0	0.301662708
TOP1MT	2	0	0.277899344
PLEC	2	0	0.399371069
PNMT	2	0	0.327319588
CTRC	2	0.015748031	0.315136476
CCKAR	2	0	0.299528302
PKN1	2	0	0.333333333
FADS1	2	1.17E-04	0.32987013
AHR	2	0	0.335978836
SLC22A12	1	0	0.318295739
POLB	1	0	0.218213058
MGAM2	1	0	0.246601942
CPT2	1	0	0.314356436
CPA1	1	0	0.240075614
CCKBR	1	0	0.296037296
CES2	0	-	-
ICMT	0	-	-
SLC13A5	0	-	-

Table S5. The information of top 30 signaling pathway.

Pathway	Fold Enrichment	*P*-Value	Genes enrichment in the pathway
Phenylalanine metabolism	15	0.016	MIF, MAOA, MAOB
Arachidonic acid metabolism	11.8	0.00000071	ALOX15, ALOX5, HPGDS, LTA4H, PLA2G2A, PTGES2, PTGES, PTGS1, PTGS2
African trypanosomiasis	10.8	0.0011	IL1B, IL6, PRKCA, PRKCB, SELE
Antifolate resistance	10.7	0.0058	DHFR2, IL1B, IL6, NFKB1
Morphine addiction	9.7	0.00000015	ADORA1, PDE10A, PDE11A, PDE1B, PDE3B, PDE4A, PDE4B, PDE4D, PDE7A, PRKCA, PRKCB
AGE-RAGE signaling pathway in diabetic complications	9.6	0.000000034	NOX4, IL1B, IL6, NFKB1, PRKCA, PRKCB, PRKCD, PRKCE, SELE, STAT3, STAT5A, VEGFC
Tyrosine metabolism	8.9	0.0097	MIF, MAOA, MAOB, TYR
Purine metabolism	8.1	0.000000049	ADA, ADK, PDE10A, PDE11A, PDE1B, PDE3B, PDE4A, PDE4B, PDE4D, PDE5A, PDE7A, PNP, XDH
Amphetamine addiction	7	0.0016	HDAC1, HDAC2, MAOA, MAOB, PRKCA, PRKCB
Regulation of lipolysis in adipocytes	6.9	0.0056	ADORA1, MGLL, PDE3B, PTGS1, PTGS2
Inflammatory mediator regulation of TRP channels	6.5	0.0002	IL1B, PRKCA, PRKCB, PRKCD, PRKCE, TRPA1, TRPM8, TRPV3
Inflammatory bowel disease	6.2	0.0084	IL1B, IL2, IL6, NFKB1, STAT3
Graft-versus-host disease	5.7	0.095	CFD, IL1B, IL2, IL6, NFKB1, PRKCA, PRKCB, STAT3
TNF signaling pathway	5.6	0.0005	IL1B, IL6, MAP2K4, MAP3K8, NFKB1, PTGS2, SELE, VEGFC
Serotonergic synapse	5.6	0.00053	ALOX15, ALOX5, MAOA, MAOB, PTGS1, PTGS2, PRKCA, PRKCB
Neutrophil extracellular trap formation	5.5	0.0000037	CTSG, FPR1, HDAC1, HDAC10, HDAC2, HDAC3, HDAC4, HDAC6, HDAC9, MPO, NFKB1, PRKCA, PRKCB
Amoebiasis	5.5	0.0016	CTSG, IL1B, IL6, NOS2, NFKB1, PRKCA, PRKCB
Parathyroid hormone synthesis, secretion and action	5.3	0.0019	CYP27B1, FGFR1, PDE4A, PDE4B, PDE4D, PRKCA, PRKCB
Th17 cell differentiation	5.2	0.0021	AHR, IL1B, IL2, IL6, NFKB1, STAT3, STAT5A

Leishmaniasis	5.2	0.015	IL1B, NOS2, NFKB1, PTGS2, PRKCB
Drug metabolism - other enzymes	5	0.017	GSTM1, MPO, TPMT, TYMP, XDH
Acute myeloid leukemia	4.8	0.05	MPO, NFKB1, STAT3, STAT5A
Alcoholism	4.7	0.0001	ADORA2A, ADORA2B, HDAC1, HDAC10, HDAC2, HDAC3, HDAC4, HDAC6, HDAC9, MAOA, MAOB
Chagas disease	4.7	0.0085	IL1B, IL2, IL6, MAP2K4, NOS2, NFKB1
Nucleotide metabolism	4.7	0.021	ADA, ADK, PNP, TYMP, XDH
Thyroid hormone signaling pathway	4.6	0.0038	BMP4, HDAC1, HDAC2, HDAC3, PRKCA, PRKCB, THRA
C-type lectin receptor signaling pathway	4.6	0.0092	IL1B, IL2, IL6, NFKB1, PTGS2, PRKCD
Chemical carcinogenesis - DNA adducts	4.6	0.055	EPHX1, GSTM1, HPGDS, PTGS2
Insulin resistance	4.5	0.011	IL6, NFKB1, PRKCB, PRKCD, PRKCE, STAT3

Table S6. Molecular docking of key targets.

Compounds	Binding energy (ΔG_{bind}, kcal/mol)			
	**TNF (PDB: 2AZ5)**	**IL6 (PDB: 1ALU)**	**IL1B (PDB: 5I1B)**	**PTGS2 (PDB: 3LN1)**
**Phytochemicals**				
(+)-Vouacapenic acid	-8.9	-7.3	-6.4	-7.8
4-*O*-Methylgallic acid	-5.5	-4.9	-5.3	-6.5
5, 7-Dihydroxy-6-oxoheptadecanoic acid	-5.1	-4.2	-4.1	-6.5
Apigenin	-7.5	-6.8	-7.1	-9.8
Augustic acid	-9.1	-7.4	-7.4	-8.5
Azadirachtin	-6.3	-5	-5.7	-7.3
Berberine	-8.7	-7.4	-6.9	-7.9
Bergapten	-6.5	-5.5	-5.9	-8.1
Bergenin	-7.3	-5.8	-6.7	-7.2
Butylidenephthalide	-6.6	-5.6	-6	-7.9
Caffeic acid	-5.7	-4.9	-6.1	-7.2
Chrysophanol	-8	-6.4	-7.4	-9.4

Citreorosein	-7.5	-6.4	-6.8	-8.9
Cnidilide	-6.5	-5.3	-5.5	-7.5
Columbin	-8.7	-7.1	-7.1	-7.4
Diosmetin	-7.6	-6.9	-7.2	-8.9
Emodin	-7.6	-6.7	-7.2	-9.1
Ethyl gallate	-5.5	-4.6	-5.2	-6.3
Eugenol	-5.8	-4.6	-5.5	-6.3
Ferulic acid	-5.7	-4.8	-6.2	-7.3
Gallic acid	-5.6	-5	-6.4	-6.4
Genkwanin	-7.6	-6.5	-7.1	-9.1
Harperamone	-6.6	-5.1	-5.8	-8
Higenamine	-7.3	-6.1	-6.9	-8.8
Hispidulin	-7.4	-6.9	-7	-9.4
Isorhamnetin	-7.6	-6.7	-7.4	-9.7
Isoscopoletin	-6	-5.2	-5.6	-7.4
Jatrorrhizine	-7.7	-6.9	-6.8	-7
Kaempferol	-7.3	-6.5	-7.2	-9.5
Ligustilide	-6.4	-5.2	-5.8	-7.5
Linalool	-5	-4.6	-4.5	-6.1
Loureirin A	-6.6	-5.8	-5.8	-8.1
Loureirin B	-6.7	-5.6	-5.8	-8.1
Loureirin C	-6.7	-6.3	-6.5	-8.5
Luteolin	-7.7	-7.2	-7.5	-9.7
Magnoflorine	-7.9	-6.4	-6.9	-8.8
Methyl gallate	-5.8	-4.8	-5.1	-6.3
Nimbin	-7.5	-6.3	-6.5	-7.2
Nimbolide	-9.1	-7.3	-7	-8.2
N-trans-feruloyltyramine	-7	-6.4	-6.8	-8.2
Obacunone	-9.1	-7.7	-8.2	-8.8
O-Methylalloptaeroxylin	-7.4	-6.3	-6.4	-8.9
Palmatine	-8	-6.6	-6.5	-6.7
p-Coumaric acid	-5.7	-4.8	-5.9	-6.9
Pectolinarigenin	-7.5	-6.5	-6.8	-8.6
Perforatic acid	-7.5	-6.4	-6.6	-8.9

Perforatic acid methyl ester	-7.4	-6.4	-6.5	-9.3
Peucenin-7-methyl ester	-6.5	-5.6	-5.9	-8.3
Phloretin	-6.9	-6.2	-6.4	-8.3
Physcion	-7.5	-6.2	-6.8	-8.9
Protocatechuic acid	-5.6	-4.9	-5.6	-6.4
Pterostilbene	-6.6	-6.1	-6.3	-8.5
Quercetin	-7.2	-7.1	-7.5	-9.6
Resveratrol	-6.7	-6	-6.5	-8.9
Rhein	-8.3	-6.6	-7.3	-9.2
Salsolinol	-6.4	-5.2	-5.8	-7.1
Secoisolariciresinol	-6.3	-4.8	-6.2	-7.6
Spathulenol	-6.9	-5.4	-6.4	-7
Stigmasterol glucoside	-8.9	-6.9	-7.4	-8.5
Syringaresinol	-7	-6.1	-6.3	-7.1
Tinosporol C	-6.3	-5.2	-5.5	-7.4
Tyramine	-5.1	-4.1	-5.1	-5.7
Vanillic acid	-5.3	-4.6	-5.8	-6.5
Ziganein	-6.2	-4.4	-5.3	-7.1
**Drug standard**				
Thalidomide	-7.4	-	-	-
Tofacitinib	-	-6.4	-	-
Anakinra	-	-	-6.6	-
Celecoxib	-	-	-	-12.1

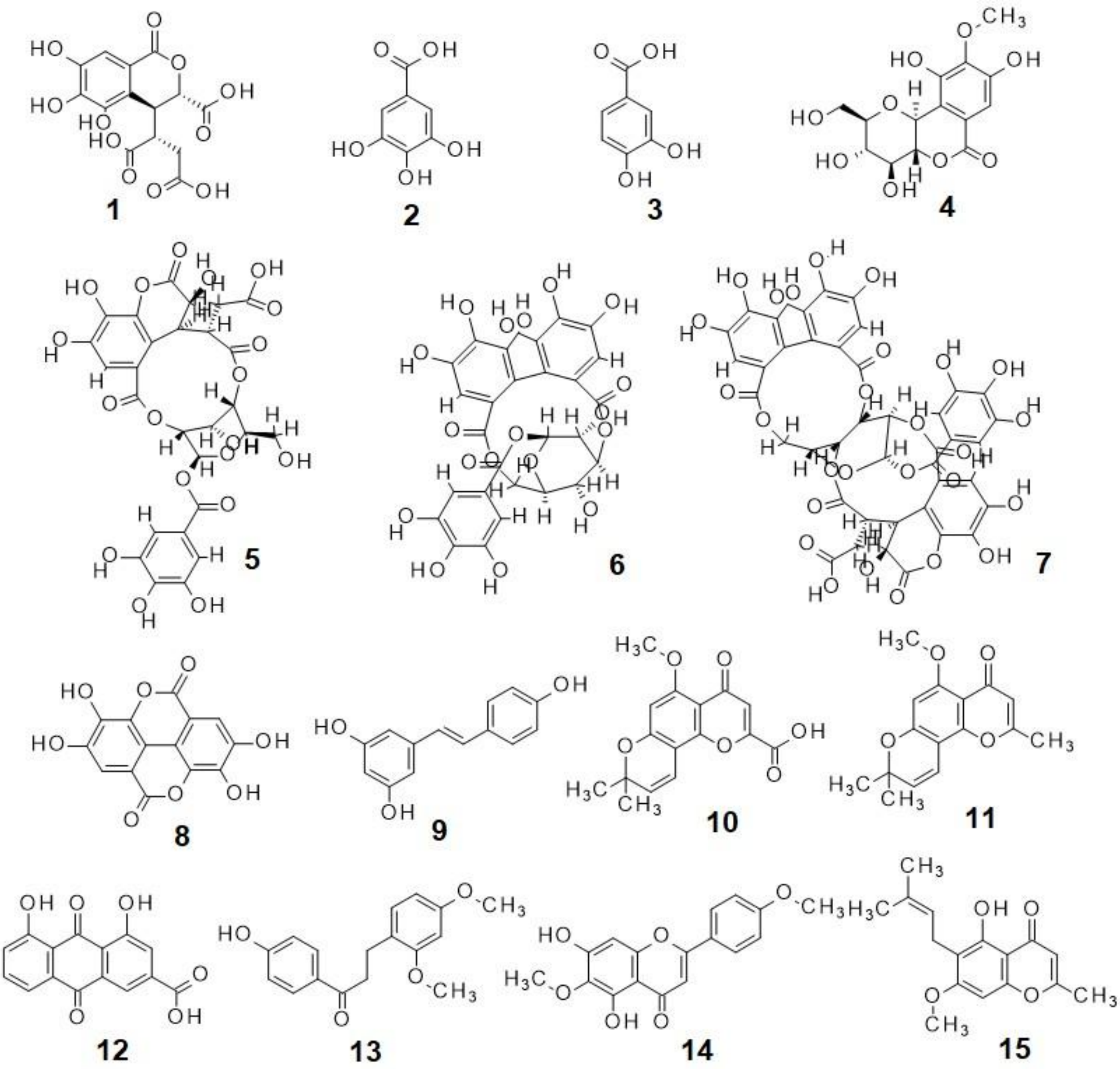

Figure S1. Structures of markers for MHR extract: chebulic acid (**1**), gallic acid (**2**), protocatechuic acid (**3**), bergenin (**4**), chebulanin (**5**), corilagin (**6**), chebulagic acid (**7**), ellagic acid (**8**), resveratrol (**9**), perforatic acid (**10**), *O*-methyllaloptaeroxyrin (**11**), rhein (**12**), loureirin A (**13**), pectolinarigenin (**14**) and peucenin-7-methyl ether (**15**).

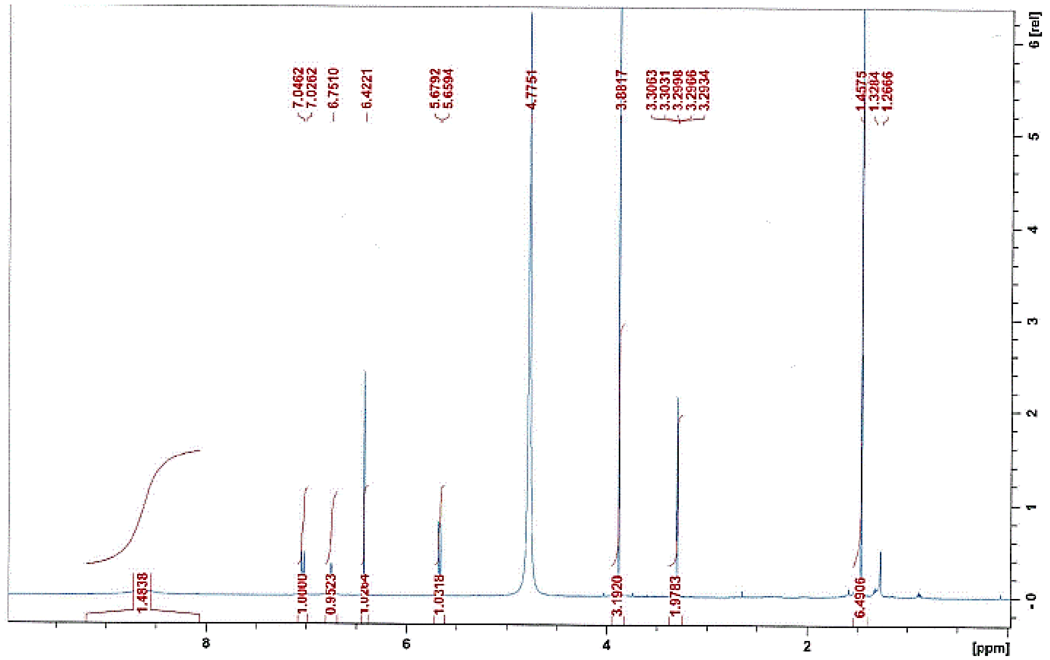

Figure S2. ^{1}H NMR spectrum (400 MHz, CD_3OD) of perforatic acid (**10**).

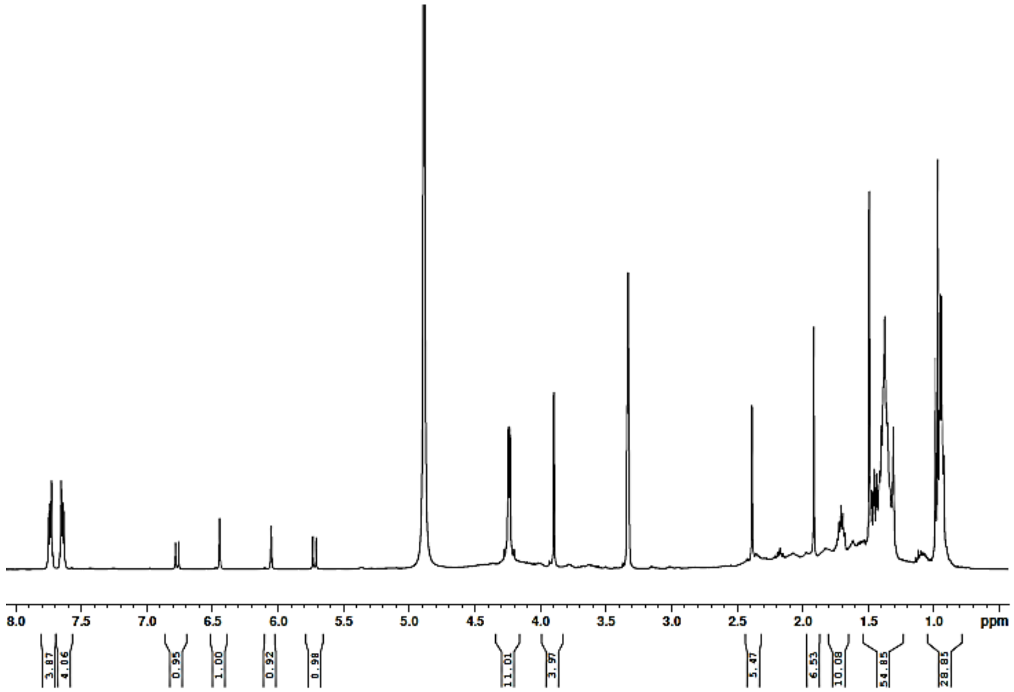

Figure S3. ^{1}H NMR spectrum (400 MHz, CD_3OD) of *O*-methyllaloptaeroxyrin (**11**).

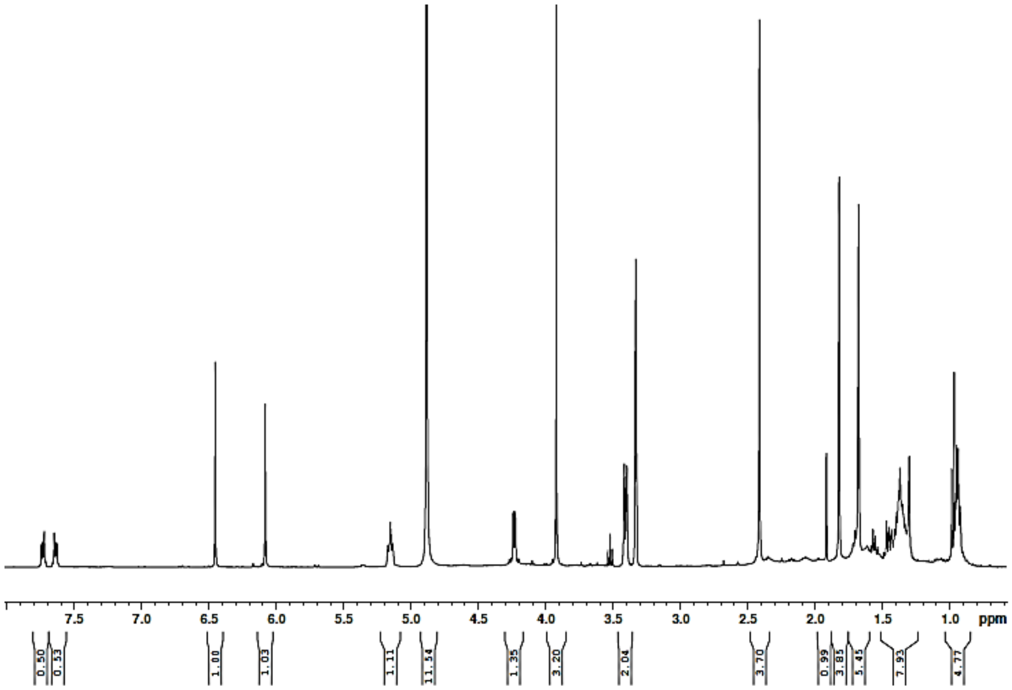

Figure S4. ^{1}H NMR spectrum (400 MHz, CD_3OD) of peucenin-7-methyl ether (**15**)
